# Exploring the Role of Glycine Metabolism in Coronary Artery Disease: Insights from Human Genetics and Mouse Models

**DOI:** 10.3390/nu17010198

**Published:** 2025-01-06

**Authors:** Subarna Biswas, James R. Hilser, Nicholas C. Woodward, Zeneng Wang, Janet Gukasyan, Ina Nemet, William S. Schwartzman, Pin Huang, Yi Han, Zachary Fouladian, Sarada Charugundla, Neal J. Spencer, Calvin Pan, W. H. Wilson Tang, Aldons J. Lusis, Stanley L. Hazen, Jaana A. Hartiala, Hooman Allayee

**Affiliations:** 1 Department of Surgery, Keck School of Medicine, University of Southern California, Los Angeles, CA 90033, USA; 2 Department of Population and Public Health Sciences, Keck School of Medicine, University of Southern California, Los Angeles, CA 90033, USA; 3 Department of Biochemistry and Molecular Medicine, Keck School of Medicine, University of Southern California, Los Angeles, CA 90033, USA; 4 Department of Cardiovascular and Metabolic Sciences, Lerner Research Institute, Cleveland Clinic, Cleveland, OH 44195, USA; 5 Center for Microbiome and Human Health, Cleveland Clinic, Cleveland, OH 44195, USA; 6 Department of Cardiovascular Medicine, Heart, Vascular and Thoracic Institute, Cleveland Clinic, Cleveland, OH 44195, USA; 7 Department of Medicine, David Geffen School of Medicine of UCLA, Los Angeles, CA 90095, USA; 8 Department of Human Genetics, David Geffen School of Medicine of UCLA, Los Angeles, CA 90095, USA; 9 Department of Microbiology, Immunology, and Molecular Genetics, David Geffen School of Medicine of UCLA, Los Angeles, CA 90095, USA

**Keywords:** glycine, coronary artery disease, atherosclerosis, genome-wide association study, Mendelian randomization, dietary supplementation, mice

## Abstract

**Background:** Circulating glycine levels have been associated with reduced risk of coronary artery disease (CAD) in humans but these associations have not been observed in all studies. We evaluated whether the relationship between glycine levels and atherosclerosis was causal using genetic analyses in humans and feeding studies in mice. **Methods:** Serum glycine levels were evaluated for association with risk of CAD in the UK Biobank. Genetic determinants of glycine levels were identified through a genome-wide association study (GWAS) and used to evaluate the causal relationship between glycine and risk of CAD by Mendelian randomization (MR). A dietary supplementation study was carried out with atherosclerosis-prone apolipoprotein E deficient (*ApoE^−/−^*) mice to determine the effects of increased circulating glycine levels on cardiometabolic traits and aortic lesion formation. **Results:** Among 105,718 UK Biobank subjects, elevated serum glycine levels were associated with significantly reduced risk of prevalent CAD (Quintile 5 vs. Quintile 1 OR = 0.76, 95% CI 0.67–0.87; *p* < 0.0001) and incident CAD (Quintile 5 vs. Quintile 1 HR = 0.70, 95% CI 0.65–0.77; *p* < 0.0001) after adjustment for age, sex, ethnicity, anti-hypertensive and lipid-lowering medications, blood pressure, kidney function, and diabetes. A GWAS meta-analysis with 230,947 subjects identified 61 loci for glycine levels, of which 26 were novel. MR analyses provided modest evidence that genetically elevated glycine levels were causally associated with reduced systolic blood pressure and risk of type 2 diabetes, but did not provide significant evidence for an association with decreased risk of CAD. Glycine supplementation in mice had no effects on cardiometabolic traits or atherosclerotic lesion development. **Conclusions:** While expanding the genetic architecture of glycine metabolism, MR analyses and in vivo feeding studies did not provide evidence that the clinical association of this amino acid with atherosclerosis represents a causal relationship.

## 1. Introduction

Atherosclerotic coronary artery disease (CAD) is a complex, multi-factorial process characterized by the accumulation of lipids and fibrous elements in the arterial walls [1]. Epidemiological and genetic studies have established traditional risk factors, such as elevated blood pressure and plasma lipid levels, as causal drivers of CAD pathogenesis. A large body of evidence has also implicated lipid oxidation and inflammation at the level of the vessel as important mediators of atherosclerosis [2]. For example, low-density lipoprotein (LDL) particles that become trapped in the sub-endothelial space become oxidized and promote the recruitment of monocytes into the vessel wall where they differentiate into macrophages and give rise to cholesterol engorged “foam cells” [3]. Despite this knowledge, many of the underlying causal biological mechanisms for atherosclerosis remain unknown [1]. For example, of the ~300 genetic risk loci identified for CAD, only one-third harbor genes known to be involved in lipid metabolism or blood pressure regulation [4,5]. Furthermore, lipid lowering therapies and anti-hypertensive medications are only partially effective in reducing CAD risk, and more than 50% of patients with an acute event do not exhibit these traditional risk factors [6]. Thus, there is a critical need to identify other mechanisms underlying CAD pathogenesis.

Integrating metabolomics with genetic and clinical data can offer a window into the intricate pathways governing the complex pathophysiology underlying atherosclerosis [7]. This approach has previously implicated glycine–a simple amino acid–as a potential factor in modulating risk of CAD [8,9]. For example, dietary glycine supplementation in humans and rodents has been shown to reduce blood pressure [10,11], improve insulin-sensitivity [12], and diminish platelet aggregation [13,14], thus providing plausible interconnected mechanisms for the protective associations observed between glycine levels and cardiovascular risk. However, recent genetic studies have provided conflicting evidence regarding a causal role of glycine in atherosclerosis [15,16,17,18]. One potential explanation for these discrepancies may be due to the pleiotropic associations that several of the genetic variants identified for glycine levels exhibit with other CAD-related traits, including visceral adipose tissue accumulation [19] and type 2 diabetes [20]. Thus, Mendelian randomization (MR) approaches that use variants with pleiotropic associations would violate one of the central assumptions of MR. Other studies have postulated that lower glycine is a sequela of other CAD-related disease processes, such as insulin resistance [21], obesity-related increases in branched-chain amino acids (BCAAs) [22], or decreased glycine synthesis [23]. Consequently, it remains unclear whether the epidemiological associations observed between higher glycine levels and reduced risk of CAD represents a true inverse causal relationship with translatable potential. In the present study, we used complementary and physiologically relevant clinical and genetic in humans and dietary supplementation strategies in mice to comprehensively evaluate the causal association of glycine with cardiovascular risk.

## 2. Materials and Methods

### 2.1. Study Populations

The UK Biobank is a large, multi-site cohort that recruited participants between 40–69 years of age who were registered with a general practitioner of the UK National Health Service (NHS) [24]. Between 2006–2010, a total of 503,325 individuals were enrolled through 22 assessment centers in the UK. At enrollment, extensive data on demographics, ethnicity, education, lifestyle indicators, imaging of the body and brain, and disease-related outcomes were obtained through questionnaires, health records, and/or clinical evaluations. Blood samples were also collected at baseline for measurement of serum biomarkers that are either established disease risk factors or routinely measured as part of clinical evaluations. All study participants provided informed consent and the study was approved by the North West Multi-Centre Research Ethics Committee. The present analyses with the UK Biobank were approved by the Institutional Review Board of the USC Keck School of Medicine (Study ID: HS-17-00920; approved 21 November 2017).

The Cleveland Clinic GeneBank study is a single site sample repository generated from ~10,000 consecutive patients undergoing elective diagnostic coronary angiography or elective cardiac computed tomographic angiography with extensive clinical and laboratory characterization and longitudinal observation (https://clinicaltrials.gov/ct2/show/NCT00590200; accessed on 31 December 2024). Subject recruitment occurred between 2001 and 2007. Ethnicity was self-reported and information regarding demographics, medical history, and medication use was obtained by patient interviews and confirmed by chart reviews. All clinical outcome data were verified by source documentation. CAD was defined as adjudicated diagnoses of stable or unstable angina, myocardial infarction (MI) (adjudicated definition based on defined electrocardiographic changes or elevated cardiac enzymes), angiographic evidence of ≥50% stenosis of one or more major epicardial vessel, and/or a history of known CAD (documented MI, CAD, or history of revascularization). Plasma glycine levels at enrollment were quantified in 3037 subjects of northern European ancestry in three batches (n = 384, 882, and 1771) by stable isotope dilution high performance liquid chromatography with online electrospray ionization tandem mass spectrometry (LC/MS), as described previously [25]. The GeneBank cohort has been used previously for discovery and replication of novel genes and risk factors for atherosclerotic disease [15,25,26,27,28,29,30,31]. Written informed consent was obtained from all participants prior to enrollment. The GeneBank study has been approved by the Institutional Review Board of the Cleveland Clinic and the present analyses were approved by the Institutional Review Board of USC Keck School of Medicine (Study ID: 055013; approved 6 June 2005).

### 2.2. Clinical Definitions in UK Biobank

All subjects in the UK Biobank with serum glycine levels were included in the clinical analyses with CAD regardless of ancestry. CAD cases were defined as having been assigned ICD10 codes I21, I22, I23, I25.2, I24.0, I24.8, I24.9, I25.0, I25.1, I25.4, I25.8, or I25.9, which included doctor-diagnosed and self-reported ischemic heart disease (Data-Fields 6150 and 20002, respectively) on or before the date of enrollment (Data-Field 53). Time to incident CAD was derived by calculating the number of days from the date when the participant without a prior CAD diagnosis attended the baseline assessment (Date-Field 53) to the date on which the first ICD10 code for CAD was assigned to the subject (Date-Field 41262 and 41280). Type 2 diabetes (T2D) status was defined based on ICD10 code E11 and kidney function was based on estimated glomerular filtration rate (eGFR), as calculated using a standardized formula [32] with serum creatinine, age, sex, and self-reported ethnic background (Data-Field 21000). Antihypertensive and lipid-lowering medication use were also based on self-reported data (Data-Fields 6153, 6177 and 20003).

### 2.3. Clinical Analyses in UK Biobank

Glycine levels were derived from nuclear magnetic resonance (NMR)-based metabolomics analyses that were carried out on serum samples at the baseline blood draw from a random subset of ~121,000 UK Biobank subjects [33]. Serum glycine levels were categorized into quintiles and evaluated for association with risk of prevalent CAD by logistic regression. This analysis included 101,619 controls and 4099 cases with CAD at the time of enrollment in the UK Biobank and the model was adjusted for age, sex, self-reported ethnicity, anti-hypertensive medications, lipid-lowering medications, eGFR, and systolic blood pressure. Cox proportional hazards regression models were used to evaluate quintiles of glycine levels with incident risk of CAD with adjustment for age, sex, self-reported ethnicity, anti-hypertensive medications, lipid-lowering medications, and T2D at baseline. Association of serum glycine levels with risk incident CAD was evaluated only among participants who were defined as controls in the prevalent CAD logistic regression analysis and for whom complete data were available (n = 101,608). Incident CAD was defined based on participants who were assigned an ICD10 code for CAD after enrollment into UK Biobank and up to 31 October 2022 (maximum follow-up period was capped at 5000 days). Participants assigned an ICD10 code for CAD within 14 days of enrollment into UK Biobank were excluded and those who died from causes not classified under ICD10 codes for CAD were censored at the time of death. Trend and quintile comparison *p*-values were calculated for both logistic and time-to-event analyses. All statistical analyses were carried out with SAS 9.4 (SAS Institute, Inc., Cary, NC, USA).

### 2.4. GWAS for Circulating Glycine Levels in the Genebank and UK Biobank Cohorts

Genome-wide genotyping in GeneBank was carried out with either the Affymetrix Genome-Wide Human Array 6.0 Chip (n = 3031) or the Illumina Infinium Global Screening Array-24 v2.0 (GSA) BeadChip (n = 1728). Prior to imputation, genomic coordinates of SNPs on each genotyping platform were first converted to GRCh37/hg19. Quality control steps included removal of duplicate SNPs as well as those with call rates < 97%, minor allele frequencies (MAFs) < 1%, and without chromosome and base pair position. Individuals with genotype call rates < 90%, of African American ancestry, and outliers from PCA analysis were also excluded, resulting in 671,968 SNPs in 2972 participants genotyped with the Affymetrix 6.0 Chip, and 539,533 SNPs in 1624 participants genotyped with the GSA Chip. Imputation was carried out for unmeasured SNPs on the forward (+) strand using 1000 Genomes Project (Phase 3 release, v5) and Haplotype Reference Consortium (vr1.1 2016) as reference panels through the University of Michigan Imputation Server (https://imputationserver.sph.umich.edu; accessed on 31 December 2024). After imputation, subjects with discordant sex were excluded and SNPs that had Hardy–Weinberg equilibrium *p*-values < 0.0001, were duplicates or multiallelic, had imputation quality scores < 0.3, and with MAFs < 1% were removed. This resulted in 9,185,470 SNPs available for analysis in 3037 GeneBank subjects with plasma glycine measurements. Since all subjects the Genebank cohort were of European ancestry, circulating glycine levels were analyzed by linear regression with adjustment for age, sex, and genotyping array as implemented in PLINK (v1.9) (http://www.cog-genomics.org/plink/1.9/; accessed on 31 December 2024) [34].

In UK Biobank, quality control of samples, DNA variants, and imputation were performed by the Wellcome Trust Centre for Human Genetics [24]. Briefly, ~90 million SNPs imputed from the Haplotype Reference Consortium, UK10K, and 1000 Genomes imputation were available in UK Biobank. After filtering on autosomal SNPs with INFO scores > 0.8 (directly from the UK Biobank) and with MAFs  > 1%, 9,560,226 variants were available in 117,152 UK Biobank subjects with serum glycine measurements. Since all participants in the UK Biobank were included in the GWAS analysis for glycine levels, a standard (infinitesimal) linear mixed model (LMM) was used to correct for population structure due to relatedness and ancestral heterogeneity. Analyses were carried out with BOLT-LMM (v2.3.4) and included adjustment for age, sex, the first 20 principal components, and genotyping array [35]. The genome-wide significance thresholds for GWAS analyses in the GeneBank and UK Biobank cohorts were set at *p* = 5.0 × 10^−8^.

### 2.5. Meta-Analysis for Circulating Glycine Levels

A meta-analysis for circulating glycine levels was carried out by combining the GWAS results generated in GeneBank and UK Biobank with publicly available summary statistics from 13 previously published datasets [15,36,37,38,39,40,41]. In total, 230,947 multi-ancestry subjects from 13 datasets were included in the meta-analysis (Supplementary Table S2). Given the differences in metabolomics platforms used, different GWAS analytical approaches, and the lack of uniform effect estimates across these datasets, we used a weighted Z-score method for the meta-analysis for glycine levels. GWAS summary statistics from Lotta et al. [40] were first imputed with summary statistics imputation (SSimp) (v0.5.6) software [42] using the European population from the 1000 Genomes Project (Phase 3 release, v5) as a reference panel for LD computation. After filtering on autosomal SNPs with MAF  > 1%, this imputation resulted in Z-scores and *p*-values for association of 7,667,668 SNPs with circulating glycine levels in the cohorts used by Lotta et al. [40]. We next harmonized the summary GWAS data from the remaining studies to also match the data in the GeneBank and UK Biobank cohorts and converted genomic coordinates for all datasets to GRCh37/hg19. A weighted Z-score meta-analysis was performed by combining the Z-score and *p*-value summary level data for SNPs that were common to at least two datasets (Supplementary Table S1), as implemented in METAL [43]. The genome-wide threshold for significant associations in the GWAS meta-analyses was set at *p* = 5.0 × 10^−8^. Manhattan and quantile-quantile (QQ) plots were generated using the ‘qqman’ package (v0.1.4) [44]. A locus was defined as novel if the lead SNP was in weak or no linkage disequilibrium (LD; r^2^ < 0.2) with genome-wide significant variants reported previously for circulating glycine levels. The Functional Mapping and Annotation of Genome-Wide Association Studies (FUMA) program (v1.5.4) [45] was used to define independent SNPs at glycine-associated loci as those that also yielded *p* < 5.0 × 10^−8^, were at least 500 kb away from the lead variant, and were in low LD (r^2^ < 0.2) with other variants at the locus based on LD information from the 1000 Genomes Project. To determine whether loci identified for glycine levels were also associated with other clinical traits, CAD risk factors, and metabolites, a phenome-wide association study (PheWAS) was carried out using FUMA and publicly available resources, such as the GWAS Catalog (https://www.ebi.ac.uk/gwas/home; accessed on 31 December 2024), PhenoScanner [46], and the UCSC Genome Browser (https://genome.ucsc.edu/; accessed on 31 December 2024). The significance threshold for PheWAS analyses was set at *p* = 5.0 × 10^−8^ with an LD cut off of r^2^ ≥ 0.8 for proxy SNPs.

### 2.6. Genetic Risk Score Analyses

Primary level data from the UK Biobank were used to generate weighted genetic risk scores (GRS) with all 61 loci for glycine levels. For each variant, the number of alleles associated with increased glycine was multiplied by its respective effect size obtained from the GWAS analysis in the UK Biobank and summed together across all variants to generate the GRS. Association between quintiles of weighted GRS and glycine levels was tested using linear regression, with adjustment for age, sex, the first 20 principal components, and genotyping array. All analyses were carried out in R (v4.2.0) [47].

### 2.7. Mendelian Randomization (MR) Analyses

To evaluate whether glycine levels were causally associated with risk of CAD, we used the results of our GWAS analysis in the UK Biobank (since we had primary metabolomics data with absolute concentrations of glycine levels available in this large dataset) and publicly available summary results from a recently published large-scale multi-ancestry GWAS meta-analysis for CAD [4]. Effect sizes for three groups of genetic instruments for glycine levels as the exposure were taken from the GWAS results in the UK Biobank. Group 1 included variants at all 61 glycine-associated loci, Group 2 only included variants at the 6 loci that did not exhibit pleiotropic associations with other traits or metabolites, and Group 3 only included variants at the 3 non-pleiotropic loci that harbored genes involved in the glycine cleavage system. MR analyses were carried out using weighted median, inverse variance weighted, and MR Egger methods, as implemented in the “TwoSampleMR” package [48,49] for R (v4.2.0) [47]. MR analyses with the six non-pleiotropic loci were also carried out for blood pressure, body mass index (BMI), glucose/insulin traits, circulating lipid levels, and risk of T2D using effect sizes from previously published GWAS meta-analyses [50,51,52,53,54] as the outcomes.

### 2.8. Animal Husbandry and Glycine Supplementation

Animal studies were performed with approval and in accordance with the guidelines and approval of the USC Institutional Animal Care and Use Committee (IACUC) (Protocol 12006, approved 12 July 2016). All mice were housed in a temperature-controlled facility with a 12-h light/dark cycle and fed ad libitum with free access to water. Collection of blood samples on conscious mice and euthanization was carried out in accordance with USC IACUC guidelines. Male and female *ApoE^−/−^* mice (stock number: 002052) were purchased from Jackson Laboratories (Bar Harbor, Maine), bred in-house for this study, and maintained on a chow diet (PicoLab: #5053) until initiation of the glycine feeding studies. At 8–10 weeks of age, age-matched male and female *ApoE^−/−^* mice were randomly assigned to customized chow or amino acid defined diets containing either 0.3% glycine (Research Diets: A18011701, New Brunswick, NJ, USA) or 2% glycine (Research Diets: A18011702, New Brunswick, NJ, USA), respectively. Details on the composition of the two diets are provided in the Supplementary Materials.

### 2.9. Blood Measurements

Following 16 weeks of glycine supplementation, blood was collected from the submandibular vein after a 12 h overnight fast and the mice were euthanized immediately thereafter. One week prior to euthanization, a small volume (~50 μL) of non-fasting blood was also obtained in a subset of mice from the submandibular vein two hours after the beginning of the feeding cycle. After obtaining the sample, light pressure was applied to stop the bleeding and the mouse was returned back to its home cage. Absolute levels glycine and other metabolites/amino acids in plasma were quantified by LC/MS, as described above for the GeneBank cohort. Plasma total cholesterol, high-density lipoprotein (HDL) cholesterol, triglyceride, and glucose levels were determined by enzymatic colorimetric assays, as described previously [55,56]. Combined very low-density lipoprotein (VLDL) cholesterol and low-density lipoprotein (LDL) cholesterol levels were calculated by subtracting HDL cholesterol from total plasma cholesterol levels. Plasma insulin levels were measured in duplicate using Mouse Ultrasensitive Insulin ELISA kits (Alpco Inc., Salem, NH, USA). Homeostasis modeling assessment insulin resistance (HOMA-IR) was calculated according to the formula: (glucose (mmol/L) × insulin (mIU/mL)]/22.5) [57]. Inflammatory cytokines were measured using a multiplexed immunoassay kit (Meso Scale Discovery, Rockville, MD, USA) and complete blood count profiles were determined using the HEMAVET^®^ 950FS Multi-species Hematology System (Drew Scientific, Miami Lakes, FL, USA).

### 2.10. Aortic Lesion and En Face Analyses

After euthanization, hearts were perfused through the left ventricle with approximately 15 mL of phosphate-buffered saline (PBS) and characterized for atherosclerotic lesion formation, as described previously [58]. The hearts were then removed, placed in 10% formalin solution, and transferred to 30% sterile sucrose for 48hrs before being embedded in Optimal Cutting Temperature (OCT) compound (Fisher Scientific, Waltham, MA, USA). Serial interrupted 10 μm tick aortic cryosections were cut starting at the origins of the aortic valve leaflets. Every 8th section was stained with Oil Red O and hematoxylin for quantification of atherosclerotic lesion area. For each mouse, total aortic lesion size was determined in blinded fashion by summing the lesion areas of 10 sections using Image-J software, version 1.46r (NIH). En face analysis was carried out on a different set of euthanized mice after first perfusing the heart and aorta with 15 mL of PBS, followed by a formal sucrose solution (4% paraformaldehyde/7.5% sucrose/10 mM sodium phosphate buffer/2 mM EDTA/20 mM butylated hydroxytoluene) for 15 min, and rinsing again with 10 mL of PBS. Aortas from the aortic arch to iliac bifurcation were removed carefully under a microscope and the surrounding adventitial fat tissue was dissected away. The aorta was then opened longitudinally from the aortic root to iliac bifurcation and pinned on a black rubber plate filled with PBS. The aortas were then incubated in 70% ethanol for 5 min, stained with Sudan-IV solution (5 mg/mL Sudan-IV in 70% ethanol and 100% acetone) for 15 min, and de-stained with 80% ethanol for 3 min. Aortas were then briefly rinsed under running tap water to remove any residual ethanol, then submerged in PBS for image capture. Images were taken with a digital camera and Sudan-IV stained atherosclerotic lesion area was calculated using Image-J software. Lesion area along the aortic arch, descending aorta, and abdominal aorta was calculated as the percentage of total area. All image capture and quantitation for the en face analyses were conducted in a blinded fashion.

### 2.11. Statistical Analyses

Differences in measured variables between control and glycine-supplemented mice were determined by unpaired Student’s *t*-tests (Prism v6.04, GraphPad Software, Boston, MA, USA). Values are expressed as mean ± SE and differences and were considered statistically significant at *p* < 0.05 or at the significance threshold after correcting for multiple comparisons.

## 3. Results

**Figure 1:**
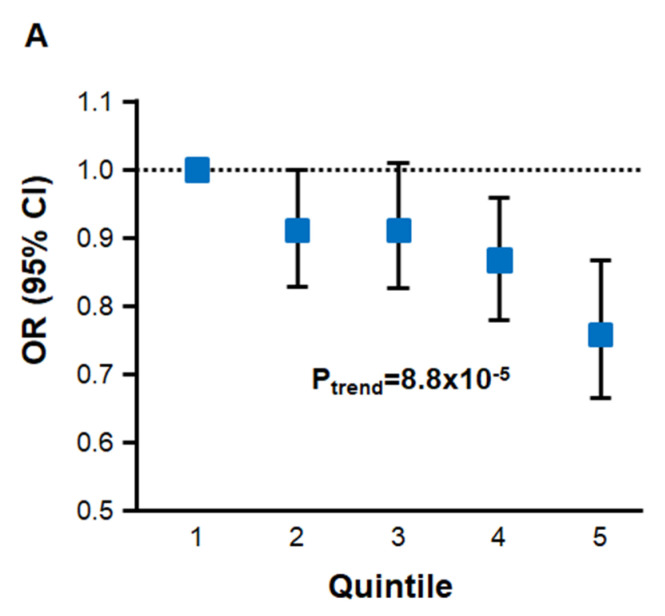

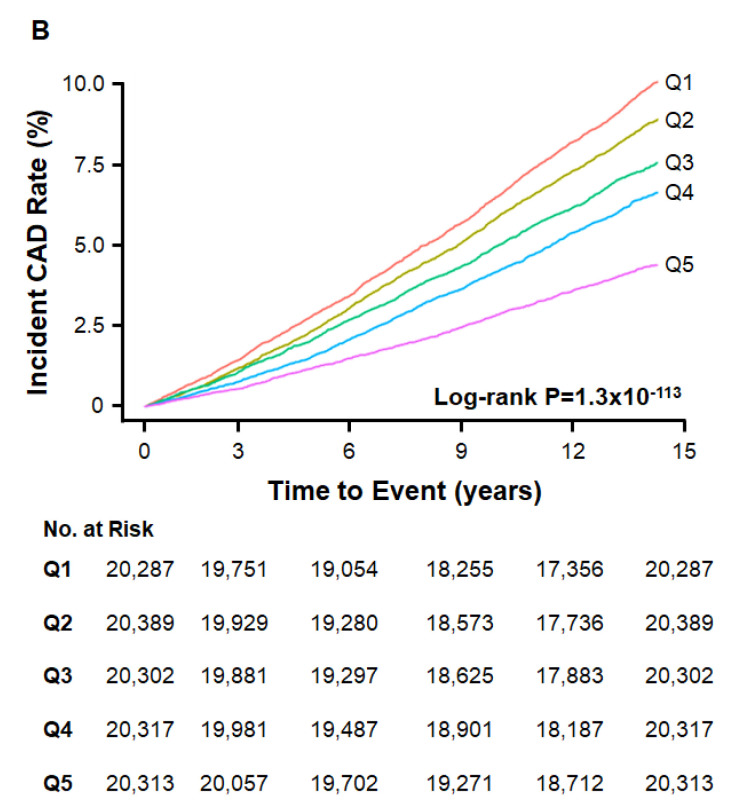
Association of Serum Glycine Levels with Risk of CAD in the UK Biobank. Individuals in the highest quintile of glycine levels had significantly reduced risk of prevalent CAD (OR = 0.76, 95% CI 0.67–0.87; *p* < 0.0001) (**A**) and incident CAD (HR = 0.70, 95% CI 0.65–0.77; *p* < 0.0001) (**B**) compared to individuals in the first quintile. *p*-value for trend for association with risk of prevalent CAD (**A**) and log-rank *p*-value for association with incident risk of CAD (**B**) across quintiles are also shown, including the number of subjects at risk of incident CAD in each quintile at baseline and the indicated follow-up time (bottom panel in **B**).

*Association of Serum Glycine Levels with Prevalent and Incident Risk of CAD in UK Biobank:* We first leveraged data from the UK Biobank to evaluate the association of circulating glycine levels with risk of CAD. The clinical characteristics of the subjects used for these analyses for whom complete clinical, demographic, and covariate data were available are shown in Supplementary Table S1. Among 105,718 subjects from the UK Biobank (4099 CAD cases/101,619 controls) with metabolomics data, higher circulating glycine levels were associated with reduced risk of prevalent CAD at the time of enrollment into UK Biobank (Table 1; Figure 1A). This atheroprotective association was particularly evident among subjects in the highest quintiles of glycine levels compared to subjects in the first quintile (OR = 0.87, 95% CI 0.78–0.96; *p* < 0.0001 for Q4 vs. Q1 and OR = 0.76, 95% CI 0.67–0.87; *p* < 0.0001 for Q5 vs. Q1) (Table 1; Figure 1A). Among the 101,608 control subjects without CAD at the time of enrollment into UK Biobank and for whom complete data were available, longitudinal analyses over 5000 days (~13 years) of follow-up revealed that higher baseline circulating glycine levels were also associated with reduced risk of incident CAD (Table 1; Figure 1B). For example, subjects in the third, fourth, and fifth quintiles of glycine levels had 13–30% reduced risk of incident CAD (*p* < 0.0001) compared to individuals with lowest levels of glycine (Table 1; Figure 1B).

*Genome-Wide Meta-Analysis for Circulating Glycine Levels:* We next carried out a large-scale GWAS meta-analysis to further define the genetic architecture of circulating glycine levels. In total, we combined GWAS summary statistics from 13 datasets comprising 230,947 multi-ancestry subjects (Supplementary Table S2).

**Figure 2:**
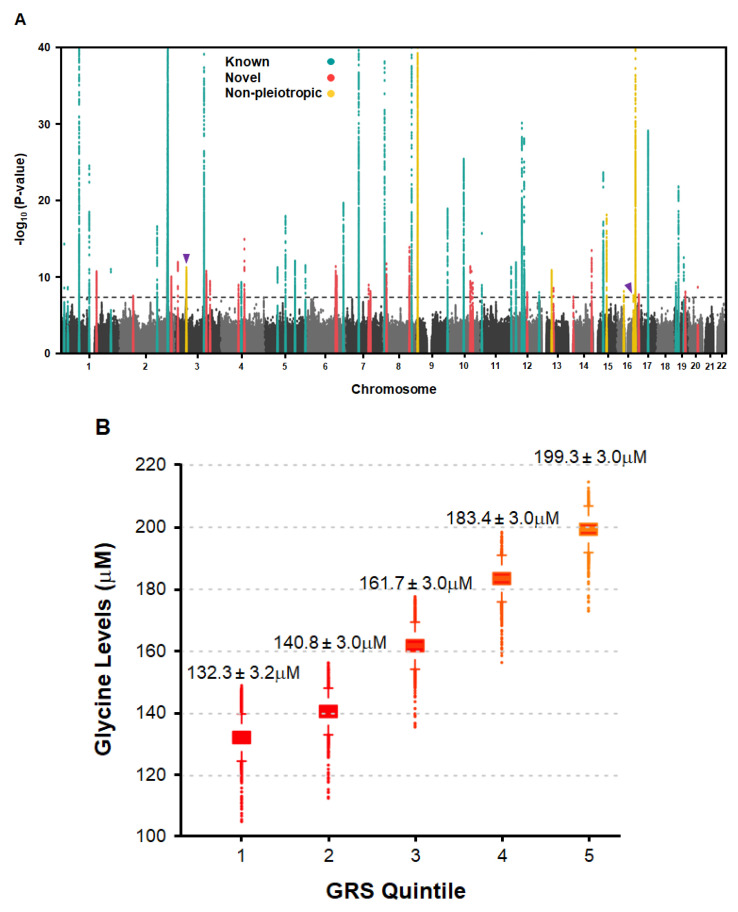
Multi-ancestry GWAS Meta-analysis and Genetic Risk Score (GRS) Analysis for Circulating Glycine Levels. (**A**) Manhattan plot shows 61 loci significantly associated with circulating glycine levels in 230,947 subjects. Novel (26) and known (35) loci are indicated by red and green dots, respectively. The seven non-pleiotropic loci are indicated by yellow dots, of which two loci on chromosomes 3 and 16 were also novel (purple arrow heads). Genome-wide thresholds for significant (*p* = 5.0 × 10^−8^) and suggestive (*p* = 5.0 × 10^−6^) association are indicated by dashed gray lines. *p*-values are truncated at −log_10_ (*p*-value) = 40. (**B**) Serum glycine levels are increased as a function of quintiles of a weighted GRS constructed with the number of glycine-raising alleles carried by individuals in the UK Biobank for the 61 loci identified in the meta-analysis (n = 23,283/quintile; total n = 116,412). Mean glycine levels are shown for each quintile (*p*-trend = 1.6 × 10^−4^).

This analysis revealed 15,230 SNPs distributed among 61 loci that were associated with circulating glycine levels at the genome-wide significance threshold (*p* = 5.0 × 10^−8^) (Figure 2A; Supplementary Table S3). Twenty-six of these loci were newly identified herein as being significantly associated with circulating glycine levels (Table 2; Supplementary Figure S1), whereas the remaining thirty-five had been identified in previous studies [15,16,59] (Supplementary Table S3). As expected, the larger sample size and power in our meta-analysis increased significance levels at many previously known loci, including *CPS1* (rs1047891; *p* = 7.8 × 10^−1101^) and *GLDC* (rs3765556; *p* = 3.9 × 10^−165^), which remained the two strongest genetic determinants of circulating glycine levels (Supplementary Table S3).

In addition, several of the 26 novel loci harbored genes were involved in glucose metabolism and insulin regulation, such as *IRS1* and *PPARG*, whereas genes localizing to other loci *(PCCB*, *CYP3A7*) are known to be involved in the metabolism of glycine and other amino acids [60,61] (Supplementary Table S3; Supplementary Figure S1). Using the absolute levels of glycine available in the UK Biobank, we also constructed a weighted genetic risk score (GRS) with all 61 SNPs, which revealed a dose-dependent increase in glycine levels as a function of carrying alleles that were associated with higher glycine (*p*-trend = 1.6 × 10^−4^). Compared to those in the bottom quintile for carrying glycine-raising alleles (range 104.2–149.0 mM), serum glycine levels were significantly increased by ~67.0 ± 3.0 mM (*p* = 8.1 × 10^−67^) among individuals in the top quintile (range 173.0–214.6 mM) (Figure 2B).

We next carried out PheWAS analyses, which revealed that 55 of the 61 loci identified for glycine levels also exhibited pleiotropic associations with levels of other circulating metabolites or known CAD risk factors, such as lipid levels and blood pressure (Supplementary Table S4). Of the remaining six loci for glycine levels, three harbored genes encoding components of the glycine cleavage system (*AMT*, *GLDC*, and *GCSH*) [62] (Supplementary Table S3; Supplementary Figure S1). Genes localizing to the remaining three loci included those involved in mitochondrial regulation of oxidative stress (*VWA8*) [63], transcription and chromosome modulation (*ZNF763)* [64], or solute transport (*AQP9*) [65]. Altogether, the lead variants at the 61 loci explained 15.6% of the variance in glycine levels, while the 6 non-pleiotropic SNPs alone explained 2.9%. Of the six non-pleiotropic variants, none were significantly associated with CAD at the Bonferroni-corrected threshold for testing six variants (*p* = 0.05/6 = 0.0083) (Supplementary Table S3).

**Figure 3:**
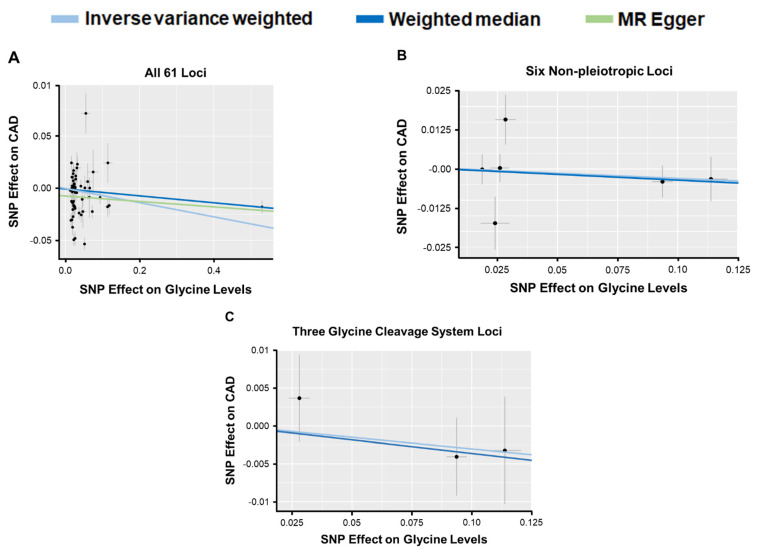
Results of MR Analyses to Evaluate Causal Association of Circulating Glycine Levels with Risk of CAD. Effect sizes of lead variants for circulating glycine levels identified in the meta-analysis (*x*-axis) are plotted against effect sizes for risk of CAD based on previously published summary statistics (*y*-axis). Slopes of the regressions are represented by the colored lines and derived from tests of MR by inverse variance weighted (light blue), weighted median (dark blue), or MR Egger (green) methods for all 61 glycine-associated loci (**A**), the 7 non-pleiotropic loci (**B**), and the 3 non-pleiotropic loci harboring genes involved in the glycine cleavage system (**C**).

*Mendelian Randomization (MR) Analysis with Glycine-Associated SNPs and Risk of CAD:* We next evaluated whether the clinical association of glycine levels with risk of CAD represented a causal relationship using various MR analyses. Variants identified from our meta-analysis for glycine levels were used as genetic instruments for the exposure and the results of recently published large-scale GWAS for CAD [4,5] were used for the outcome. MR analyses with all 61 glycine-associated SNPs yielded modest inverse associations with CAD based on inverse variance weighted (OR = 0.93, 95% CI 0.88–0.98; *p* = 0.01) and weighted median (OR = 0.96, 95% CI 0.94–0.97; *p* = 8.6 × 10^−7^) methods (Figure 3A). However, analyses with MR Egger, which takes into account pleiotropic effects of variants, did not provide evidence for a causal association between genetically increased glycine levels and risk of CAD (OR = 0.97, 95% CI 0.91–1.03; *p* = 0.28) (Figure 3A). We also carried out weighted median and inverse variance weighted MR analyses with the six non-pleiotropic SNPs, which also did not yield causal evidence for the association between glycine and risk of CAD (OR = 0.97, 95% CI 0.89–1.05; *p* = 0.39 for weighted median and OR = 0.97, 95% CI 0.88–1.07; *p* = 0.54 for inverse variance weighted) (Figure 3B). The most restrictive MR model, which only included non-pleiotropic variants at three loci harboring genes involved in glycine cleavage system, also did not reveal significant evidence for a causal association between glycine and risk of CAD (OR = 0.96, 95% CI 0.89–1.05; *p* = 0.39 for weighted median and OR = 0.97, 95% CI 0.90–1.05; *p* = 0.45 for inverse variance weighted) (Figure 3C). By comparison, MR analyses with the six non-pleiotropic loci and several CAD risk factors did provide modest evidence for an inverse causal relationship between genetically increased glycine levels and systolic blood pressure and risk of T2D, but not with diastolic blood pressure, lipid levels, or T2D-related metabolic traits (Supplementary Table S5).

**Figure 4:**
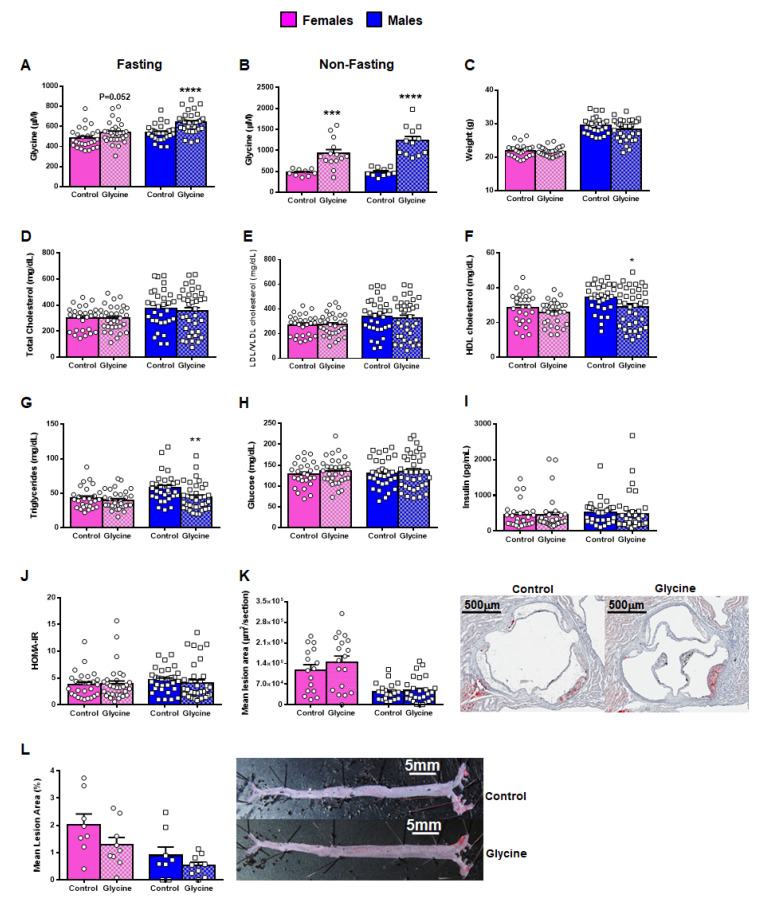
Effect of Glycine Supplementation on Plasma Cardiometabolic Traits and Atherosclerosis Development in *ApoE^−/−^* Mice. Compared to the 0.3% glycine diet (Control), mice fed the 2% glycine content diet (Glycine) had significantly higher fasting (**A**) and non-fasting (**B**) glycine levels, particularly among male mice. After 16 weeks of glycine supplementation, there were no differences in body weight (**C**) or fasting plasma levels of total cholesterol (**D**) and LDL/VLDL (**E**), whereas fasting levels of HDL cholesterol (**F**) and triglycerides (**G**) were decreased. There were also no differences with respect to metabolic traits, including fasting glucose (**H**) and insulin (**I**) levels, or HOMA-IR (**J**). Glycine supplementation did not affect atherosclerotic lesion formation assessed through serial cryosections at the aortic arch (**K**) or along the entire aorta by en face analysis (**L**). Representative sections of aortic lesions (**K**) and en face aortas stained for lipid content (**L**) are shown from female mice in the control and glycine-fed groups. Data are represented as mean ± SE. *p*-values are derived from *t*-tests carried out between control and glycine fed groups separately in males (square symbols) and females (circle symbols). * *p* < 0.05; ** *p* < 0.01; *** *p* < 0.001; **** *p* < 0.0001. n = 25–36 for data in panels (**A**,**C**–**J**); n = 9–14 for data in panel (**B**); n = 16–27 for data in panel (**K**); n = 8–9 for data in panel (**L**).

*Cardiometabolic Effects of Glycine Supplementation in Mice:* To complement the human studies, we next carried out a physiologically relevant feeding study in mice to evaluate the effect of dietary glycine supplementation on cardiometabolic traits and development of atherosclerosis (Table 3). Amino acid-defined chow diets were developed with either 2% or 0.3% glycine content that maintained nitrogen balance (Supplementary Table S6), would perturb glycine levels similar to the natural variation observed in humans, and would avoid the potential confounding effects that high fat/high cholesterol atherogenic diets could have on glycine metabolism. Given these considerations, we also selected *ApoE^−/−^* mice as the mouse model with which to carry out dietary supplementation since aortic lesions develop in this strain on a chow diet and would thus not require high fat/high cholesterol feeding. After 16 weeks, fasting glycine levels were increased in *ApoE^−/−^* mice fed the 2% glycine diet, with this elevation being highly significant in male mice and nearly significant in female mice (Figure 4A; Table 4). Non-fasting glycine levels were also robustly elevated in both male and female fed the glycine-enriched diet (Figure 4B; Table 4). Compared to the control diet, there were no significant differences in body weight, glucose and insulin-related metabolic traits, or lipid levels as a result of glycine supplementation, with the exception of a modest decrease in triglyceride levels in male mice (Figure 4C–J; Table 3). By comparison, male, but not female, *ApoE^−/−^* mice in the glycine supplementation group exhibited small differences in plasma levels of glycine-related metabolites, various amino acids, inflammatory biomarkers, and blood cell traits (Table 4; Supplementary Tables S7 and S8). However, these differences would not be considered significant given the number of traits tested. Finally, we evaluated atherosclerotic lesion area at the aortic root and along the entire aorta by en face analysis but did not observe significant differences between mice in the glycine-supplemented or control diet groups (Figure 4K,L; Table 3).

## 4. Discussion

Multiple indirect associations have suggested that glycine levels could be a protective biomarker of CAD risk. However, genetic analyses have produced equivocal evidence for a causal link between glycine and CAD, which implies that this amino acid may be correlated with other as yet unrecognized causal biomarkers, metabolites, or pathogenic mechanisms. Furthermore, studies of glycine feeding in rodents have focused on glycine-deprivation, which may not be translatable in a broader context. In the present study, we observed consistent epidemiological associations between circulating glycine levels and both prevalent and incident CAD in the UK Biobank. We also conducted the largest GWAS meta-analysis of glycine levels to date by combining summary statistics from >230,000 subjects in the UK Biobank and 12 other datasets. However, while substantially expanding our understanding of the genetic architecture of circulating glycine levels, various types of MR analyses in humans coupled with a large and well-controlled feeding experiment did not provide evidence that glycine plays a direct causal atheroprotective role in humans or mice.

Our large-scale genetic analysis identified 61 loci significantly associated with circulating glycine levels and strengthened the association signals at 35 previously known loci. Notably, the identification of 26 novel loci further revealed the diversity of metabolic pathways associated with glycine levels. For example, two novel loci harbored genes with well-known roles in glucose metabolism (*IRS1* and *PPARG*). In addition, recent studies have implicated *HNF4A* and *PROX1-AS1* in insulin resistance and T2D [66,67], whereas loci harboring *FAM13A*, *RSPO3*, and *EBPL* have been associated with body fat distribution and hepatic steatosis [68,69,70]. Genes at other loci are likely involved in the synthesis or degradation of glycine itself. For instance, *HOGA1* catalyzes the breakdown of hydroxyproline into glyoxylate, which can serve as a substrate for glycine production [71]. By comparison, *DLD* mediates the oxidation and activation of the enzyme encoded by *GCSH*, which in turn provides the substrate for the *AMT* enzyme in the oxidative cleavage of glycine [72]. *GCSH*, *DLD*, and *AMT*, together with *GLDC*, are the four components of the glycine cleavage system that metabolizes glycine into ammonia and carbon dioxide [73] for subsequent detoxification through the urea cycle. In this regard, *GLDC* was one of the most strongly associated loci for glycine levels in our meta-analysis. Moreover, arginase, encoded by *ARG1*, catalyzes conversion of arginine to ornithine in the final step of the urea cycle and is one of the major routes for removal of ammonia produced through the degradation of nitrogen-containing compounds, including glycine [74]. However, the biological mechanisms underlying association of most of the loci for circulating glycine levels remain to be determined.

Of all loci identified in our analyses, a nonsynonymous Thr1405Asn substitution (rs1047891) in the gene encoding the rate-limiting enzyme of the urea cycle, *CPS1*, remained the most significantly associated variant for glycine levels (*p* = 7.8 × 10^−1101^), consistent with prior studies [40]. The results of a recent large-scale GWAS also revealed the glycine-raising allele of rs1047891 (1405Asn) to be associated with decreased risk of CAD, although not at the genome-wide significance threshold [4]. The association of *CPS1* with risk of CAD was previously shown to be female-specific [25], similar to the sexually dimorphic association patterns observed with levels of various metabolites, including glycine, and, more recently, reduced risk of T2D in East Asians [75]. Taken together, these observations suggest that glycine may have protective cardiometabolic properties [8,15,16,74]. However, a biological mechanism for the sex-specific association of *CPS1* variants with glycine, urea cycle metabolites, and other cardiometabolic traits is not presently known and could be due to either effects of sex hormones (e.g., estrogen or testosterone) and/or genetic differences in *CPS1* expression/activity between male and females. Whether the same mechanisms underlie the sexually dimorphic effects we observed with glycine feeding in mice also remains to be determined. Furthermore, *CPS1* is one of the most pleiotropically associated loci in the genome [76] and rs1047891 has been linked to multiple other CAD risk factors, such as trimethylamine *N*-oxide (TMAO) [25], blood cell traits [77], and uric acid levels [39,78]. These observations would therefore suggest that the protective association of *CPS1* with risk of CAD could also be due to one or more of these other traits, either individually or collectively, rather than glycine per se. This notion is supported by the observation that the second most significantly associated locus for glycine levels (*GLDC*) was not associated with risk of CAD [4] or T2D [54]. By comparison, the loci we identified for glycine that were most strongly associated with CAD (*PIGV*, *TRIB1*, and *SLC22A3*) had smaller effect sizes on glycine levels than *CPS1* and *GLDC*.

Prior MR analyses in humans with sample sizes smaller than in our present study had suggested that genetically higher glycine levels were associated with reduce risk of CAD [16,18]. We also used various forms of MR with larger numbers of subjects and genetic instruments to evaluate whether glycine levels were causally associated with risk of CAD. When using lead variants at all 61 identified loci, MR analyses did yield evidence that genetically higher glycine levels were modestly associated with reduced risk of CAD. However, most of the glycine-associated loci (i.e., *CPS1*) had pleiotropic effects on other CAD-related traits, thus violating one of the fundamental assumptions of MR analysis. Therefore, we could not conclude, based on these data alone, that the atheroprotective association of glycine with CAD was attributable entirely to this amino acid. The lack of significant evidence for a causal association between genetically increased glycine levels and risk of CAD from the MR Egger analyses, which takes into account pleiotropic effects of variants by estimating causal effects after adjustment for any directional pleiotropy [79,80], further suggested that glycine may not be causally associated with risk of CAD. To address this issue, we specifically carried out MR analyses with the six glycine-associated loci for which there were no other reported associations, including the variants at three of the four loci harboring genes of the glycine cleavage complex (*AMT*, *GLDC*, and *GCSH*). However, MR analyses with both sets of non-pleiotropic variants also did not provide evidence for a causal relationship between higher glycine levels and lower risk of CAD. Collectively, these results do not provide evidence for glycine having direct cardioprotective properties. MR analyses with the six non-pleiotropic variants did suggest that genetically higher glycine levels may be associated with a modest causal effect on decreased systolic blood pressure and risk of T2D. These observations suggest that the inverse clinical associations we and others observe between glycine levels and risk of CAD could be indirect and possibly mediated through effects on peripheral metabolism and/or the vasculature.

Interestingly, a recent trans-ancestry meta-analysis in European and East Asian populations, while also not observing a causal relationship between glycine and CAD in the combined population, did find an association between glycine and several cardiovascular phenotypes, including CAD specifically in East Asians [18]. Thus, it is possible that glycine exerts protective effects in non-European ancestry groups. Moreover, several of the 61 glycine-associated loci were also linked to circulating lipid levels, obesity, and glucose metabolism. Glycine synthesis is linked to glycolytic pathways via its precursor, serine [81], while the glycine cleavage complex shares a component, dihydrolipoamide dehydrogenase (DLD), with branched-chain amino acid degradation pathways [12] that are impaired in insulin-resistance. Since glycine levels are lower in obesity and insulin-resistance [23,82], it is still possible that glycine supplementation could play a role in metabolic homeostasis and have possible metabolic benefits in certain subsets of patients.

To complement the human studies, we also carried out a comprehensive feeding study in *ApoE^−/−^* mice with isocaloric, amino acid-defined diets. Despite elevating both fasting and non-fasting glycine levels, particularly in males, the glycine-enriched diet did not reduce aortic lesion development. For the most part, glycine supplementation also did not change plasma levels of lipid and metabolic traits, inflammatory biomarkers, or blood cell parameters, particularly among female mice who are more prone to atherosclerosis than male mice. Glycine supplementation also did not induce significant changes in levels of other amino acids, including serine, threonine, and tryptophan, which have been inversely associated with risk of peripheral arterial disease [83]. Notably, our study was well-powered to detect differences in these phenotypes since, for example, there were at least ~20 mice of each sex (and in some cases even >30 mice) in most of the experimental groups, including those used to evaluate aortic lesion formation. Taken together with our human studies, these data further argue against glycine having atheroprotective properties. By comparison, a previous study had suggested that glycine deprivation in combination with a Western diet could worsen atherosclerosis in *ApoE^−/−^* mice [84]. However, this study did not observe changes in whole aortic plaque area by en face analysis or demonstrate whether glycine supplementation affected lipid levels. Furthermore, only male mice were used and there were fewer animals in each experimental group [84] relative to our study. Notably, a similar feeding experiment by the same group did observe modest effects of glycine supplementation on plasma triglyceride and total cholesterol levels but not on LDL levels on a Western diet [85]. Interestingly, other rodent feeding studies have shown improvements in insulin sensitivity with glycine supplementation, although these effects were primarily observed in the context of high fat diets as well [86]. By contrast, chow diets supplemented with glycine did not lead to metabolic changes even with longer feeding durations [87]. Our results would be consistent with these latter observations since we also used chow diets with modified glycine content. It should also be noted that the route by which glycine was delivered in feeding studies (i.e., drinking water vs. diet) can also lead to differences in observed effects.

While our results point to interesting clinical and genetic associations with glycine levels, we also note certain limitations of our study. First, glycine measurements in the UK Biobank were only available at the time of enrollment and may fluctuate over time. There may also have been residual confounding in our multivariate regression models for prevalent and incident CAD, since some risk factors remain unknown or unmeasured. In this regard, it is possible that other lifestyle factors, such as dietary habits, physical activity, and socioeconomic status, could have also been confounders in the analysis. Furthermore, our GWAS meta-analysis involved several different datasets, the majority of which were comprised of European ancestry subjects. Thus, our findings may not be generalizable to other ancestry groups, which will require additional studies with multi-ancestry datasets. Metabolomics measurements in these cohorts were also carried out on different platforms, in either serum or plasma, and at different levels of dietary intake. However, the error stemming from these variations would likely bias the results towards the null and decrease the likelihood of identifying significant genetic associations. In addition, unlike the wide variation observed in overall glycine levels in the clinical analyses, the genetic instruments used in our MR analyses, even in aggregate and when only carried out with non-pleiotropic variants, may not have provided sufficient variation in glycine levels to detect significant causal associations with risk of CAD. The use of different metabolomics platforms to quantitate glycine levels would have exacerbated this potential limitation further. By comparison, the stringent criteria we used to select genetic instruments in MR analyses resulted in evidence for causal associations between glycine and decreased systolic blood pressure and risk of T2D, consistent with prior studies [16,59]. It is also possible that our feeding study did not sufficiently elevate glycine, at least with respect to fasting levels, to reduce atherosclerosis. Thus, 16 weeks of glycine supplementation, even in *ApoE^−/−^* mice, may not have been long enough to induce differences in aortic lesion development. However, we elected to use glycine-supplemented chow diets that would lead to elevations of glycine levels that would be physiologically relevant to humans and would reduce potential confounding effects of a high fat diet. In this regard, subjects in the UK Biobank who were in the top quintile for carrying glycine-raising alleles at all 61 identified loci had glycine levels that were on average ~67 mM higher than those in the bottom quintile. In addition, the 1405 Asn variant of *CPS1*, which is the strongest genetic determinant of glycine levels in humans and is associated with reduced risk of CAD in women, increases glycine by ~50 mM per allele [25]. Thus, the elevated glycine levels observed in our feeding study with *ApoE^−/−^* mice were comparable to the effects of naturally occurring genetic variants on glycine levels in humans. However, we also note that the route by which glycine was delivered (i.e., drinking water vs. diet) is another important factor when considering the different effects reported in animal glycine supplementation studies. Finally, it is possible that the mouse model we used had inherent differences in glycine metabolism that may not fully recapitulate human metabolic conditions.

## 5. Conclusions

In summary, our study provides a more complete picture of the genetic architecture of glycine levels in humans and offers numerous opportunities to further elucidate its metabolism in mammals. While not obtaining evidence for a direct causal relationship between glycine and atherosclerosis in humans or mice, our results still suggest glycine could indirectly mitigate CAD risk, possibly through metabolic processes and/or blood pressure regulation. Future studies will be required to explore these possibilities.

## Figures and Tables

**Table 1:** Association of Serum Glycine Levels with Prevalent CAD at Time of Enrollment in UK Biobank and Incident Risk of CAD Over 5000 Days of Follow-up.

Quintile for Serum Glycine Levels	1	2	3	4	5
			Prevalent CAD		
Range (μM)	0.0–116.0	116.1–139.4	139.5–164.3	164.4–204.9	205.0–749.1
Cases/Controls	1189/19,931	980/20,189	866/20,282	679/20,461	385/20,756
OR (95% CI)	1	0.91 (0.83–1.00)	0.91 (0.83–1.01)	0.87 (0.78–0.96) **	0.76 (0.67–0.87) ***
			Incident CAD		
Range (μM)	0.0–116.5	116.6–140.0	140.1–165.1	165.2–206.2	206.3–749.1
Cases/Controls	1990/18,297	1783/18,606	1511/18,791	1322/18,995	895/19,418
HR (95% CI)	1	0.94 (0.88–1.01)	0.87 (0.81–0.92) ***	0.84 (0.78–0.90) ***	0.70 (0.65–0.77) ***

Data are shown as OR or HR with 95% CIs for association of glycine quintiles with prevalent and incident CAD, respectively, where quintile 1 is the reference group. Model for prevalent CAD is adjusted for age, sex, ethnicity, anti-hypertensive medications, lipid-lowering medications, eGFR, and systolic blood pressure. Model for incident CAD is adjusted for age, sex, ethnicity, anti-hypertensive medications, lipid-lowering medications, and T2D at baseline. ** *p* < 0.01; *** *p* < 0.0001 for comparisons to quintile 1.

**Table 2:** Novel Loci Identified for Circulating Glycine Levels.

Locus	Lead SNP	Chr	Position ^a^	Nearest Gene ^b^	EANEA	EAF	N	Z-Score	Direction ^c^	*p*-Value	*p*-Het	Beta	SE	*p*-Value ^d^
1	rs6695324	1	151,926,762	*THEM4*	A/T	0.69	222,135	−6.7	-------+--+--	2.2 × 10^−11^	0.95	0.001	0.005	0.86
2	rs1823685	2	61,435,085	*USP34*	A/G	0.17	230,887	5.5	+++---++++-?+	3.6 × 10^−8^	0.16	0.002	0.006	0.77
3	rs2943635	2	227,077,377	*IRS1*	T/C	0.69	230,735	−6.5	-+-------??--	1.1 × 10^−10^	0.41	0.032	0.005	1.6 × 10^−10^
4	rs6802898	3	12,391,207	*PPARg*	T/C	0.13	230,843	7.1	+++++-+-+?--+	1.3 × 10^−12^	0.58	−0.005	0.007	0.51
5	rs71636623	3	49,383,260	*AMT*	CAA/C	0.27	124,115	6.9	++?-++???????	6.7 × 10^−12^	0.79	0.016	0.008	0.05
6	rs4431046	3	135,849,123	*PCCB*	A/C	0.23	230,947	6.7	++-+-++-+-+++	2.0 × 10^−11^	0.72	−0.043	0.005	2.3 × 10^−15^
7	rs12492498	3	152,008,390	*MBNL1*	A/G	0.69	230,735	6.3	+++++-++-??++	4.0 × 10^−10^	0.34	0.007	0.005	0.11
8	rs9996922	4	77,437,749	*SHROOM3*	A/G	0.73	222,234	6.1	++++--?-+-??+	1.3 × 10^−9^	0.90	−0.021	0.006	7.7 × 10^−4^
9	rs13107325	4	103,188,709	*SLC39A8*	T/C	0.07	212,417	−8.0	--?++-??-???-	1.3 × 10^−15^	0.43	−0.008	0.011	0.50
10	rs7766106	6	127,455,138	*RSPO3*	T/C	0.48	230,947	−6.9	---+-+----+--	4.7 × 10^−12^	0.34	0.012	0.004	4.7 × 10^−3^
11	rs961329826	6	131,871,861	*ARG1*	G/GA	0.76	211,049	6.5	+-+-+++-????+	8.4 × 10^−11^	0.37	−0.011	0.006	0.08
12	rs45446698	7	99,332,948	*CYP3A7*	T/G	0.96	212,663	−6.1	-+?+-+-?????-	1.3 × 10^−9^	0.01	0.021	0.013	0.10
13	rs111865019	7	106,812,246	*DLD*	A/G	0.73	230,947	−5.8	---------+++-	8.3 × 10^−9^	0.99	−0.026	0.005	1.7 × 10^−7^
14	rs192322963	8	17,445,955	*SLC7A2*	A/G	0.02	208,491	7.0	+??+-+??+???+	2.1 × 10^−12^	0.12	0.017	0.019	0.38
15	rs7828742	8	116,960,729	*TRPS1*	A/G	0.40	230,779	7.7	+++++++++-??+	1.5 × 10^−14^	0.20	−0.004	0.004	0.40
16	rs3802650	10	93,577,624	*TNKS2*	A/G	0.49	170,905	−6.9	----+--+--++-	4.8 × 10^−12^	0.44	−0.007	0.005	0.12
17	rs7078003	10	99,359,412	*HOGA1*	T/C	0.16	230,843	−6.7	----+---+?-+-	2.1 × 10^−11^	0.16	0.003	0.006	0.63
18	rs11191355	10	104,392,497	*SUFU*	T/C	0.82	230,947	−6.2	---+++----++-	6.8 × 10^−10^	0.74	0.015	0.005	0.01
19	rs11177732	12	69,981,111	*CCT2*	A/G	0.22	230,947	5.7	+++++++-+-+-+	1.2 × 10^−8^	0.76	−0.002	0.005	0.70
20	rs41284816	13	50,655,989	*EBPL*	T/G	0.02	182,987	5.9	+??+-++?+???+	3.3 × 10^−9^	0.74	0.018	0.018	0.32
21	rs12889267	14	21,542,766	*ARHGEF40*	A/G	0.85	230,783	−5.5	---++--+-?+?-	4.4 × 10^−8^	0.37	−0.027	0.007	4.6 × 10^−5^
22	rs12882639	14	100,805,977	*WARS1*	T/C	0.53	230,735	−7.6	------++-??+-	3.8 × 10^−14^	0.35	−0.015	0.005	1.6 × 10^−3^
23	rs34042070	16	72,101,525	*DHX38*	C/G	0.80	216,769	5.5	+++--++++-+++	3.0 × 10^−8^	0.21	−0.040	0.005	1.3 × 10^−14^
24	rs2189338	17	5,326,341	*RPAIN*	T/C	0.40	218,825	−5.6	----+-+--?---	2.3 × 10^−8^	0.63	0.001	0.005	0.91
25	rs2340998	19	48,136,752	*ZNF541*	T/G	0.13	230,839	−5.7	---+-+---+?+-	9.8 × 10^−9^	0.10	0.017	0.006	4.6 × 10^−3^
26	rs1800961	20	43,042,364	*HNF4A*	T/C	0.03	222,832	6.0	++?-+++++???+	2.4 × 10^−9^	0.09	−0.011	0.014	0.41

^a^ SNP base pair (bp) positions are given according to NCBI build 37 of the reference human genome sequence (hg19). ^b^ Defined as gene for which lead SNP yielded expression quantitative trait locus in GTEx Project or gene closest to lead variant based on UCSC Genome Browser. ^c^ Order of cohorts for direction of effect: UK Biobank, SOL Hispanic Cohort, Tohoku Medical MegaBank Japan, GeneBank I, GeneBank II, GeneBank II, METSIM, Singapore Cohort, Canadian Longitudinal Study on Aging (CLSA) European, CLSA East Asians, CLSA South Asian, CLSA African, and meta-analysis from Lotta et al. [41]. ^d^ Association results for CAD are taken from a previously published GWAS meta-analysis [4].

**Table 3:** Effect of Dietary Glycine Supplementation on Fasting Cardiometabolic Traits and Aortic Lesion Formation in *ApoE^−/−^* Mice.

		Females			Males	
Trait	Control (n = 27)	Glycine (n = 32)	*p*-Value	Control (n = 31)	Glycine (n = 36)	*p*-Value
^a,b^ Body weight (g)	22.4 ± 0.6	22.2 ± 0.3	0.79	31.4 ± 0.9	29.7 ± 0.5	0.08
Total cholesterol (mg/dL)	299 ± 18	299 ± 17	0.99	373 ± 27	353 ± 27	0.61
LDL/VLDL cholesterol (mg/dL)	271 ± 16	273 ± 16	0.92	339 ± 26	324 ± 26	0.69
HDL cholesterol (mg/dL)	28 ± 2	26 ± 1	0.18	34 ± 1	29 ± 2	0.03
Triglycerides (mg/dL)	43 ± 3	40 ± 2	0.40	58 ± 4	44 ± 3	0.008
^c^ Glucose (mg/dL)	128 ± 6	135 ± 6	0.35	130 ± 6	134 ± 7	0.69
Insulin (pg/mL)	453 ± 68	447 ± 85	0.96	523 ± 58	481 ± 88	0.70
^d^ HOMA-IR	3.8 ± 0.5	3.9 ± 0.6	0.88	4.6 ± 0.4	4.1 ± 0.6	0.50
^e,f^ Aortic lesions (μm^2^/section)	118,060 ± 18,477	144,598 ± 21,357	0.36	44,414 ± 5967	48,423 ± 8097	0.70
^g^ En Face lesions (%)	2.0 ± 0.4	1.3 ± 0.3	0.14	0.9 ± 0.3	0.5 ± 0.1	0.26

^a^ Body weight data in females on the control and glycine diets are from 25 and 30 mice, respectively. ^b^ Body weights data in males on the control and glycine diets are from 28 and 33 mice, respectively. ^c^ Glucose levels in male mice on the control diet are from 30 animals. ^d^ Calculated according to the formula: [glucose mg/dL) × insulin (μIU/mL)]/405. ^e^ Aortic lesion data in females on the control and glycine diets are from 16 and 18 mice, respectively. ^f^ Aortic lesion data in males on the control and glycine diets are from 22 and 27 mice, respectively. ^g^ *En face* lesion data are from 8 mice of each sex on the control diet and 9 mice of each sex on the glycine diet.

**Table 4:** Effect of Dietary Glycine Supplementation on Plasma Levels of Glycine-related Metabolites and Amino Acids in *ApoE^−/−^* Mice.

	Fasting						Non-Fasting					
	Females			Males			Females			Males		
Metabolite (mM)	Control (n = 27)	Glycine (n = 32)	*p*-Value	Control (n = 30)	Glycine (n = 36)	*p*-Value	Control (n = 9)	Glycine (n = 14)	*p*-Value	Control (n = 10)	Glycine (n = 12)	*p*-Value
Choline	22 ± 1	21 ± 1	0.47	22 ± 1	22 ± 1	0.73	12.6 ± 1.1	12.2 ± 0.8	0.76	13.1 ± 1.5	11.3 ± 1.1	0.32
TMAO	2.8 ± 0.4	2.9 ± 0.3	0.87	1.0 ± 0.1	1.1 ± 0.1	0.69	5.5 ± 0.5	9.5 ± 1.6	0.06	2.2 ± 0.3	2.6 ± 0.2	0.35
Betaine	49 ± 3	50 ± 3	0.88	43 ± 3	45 ± 3	0.73	46 ± 3	47 ± 4	0.77	37 ± 5	23 ± 1	0.01
Dimethylglycine	8.8 ± 0.3	9.1 ± 0.4	0.52	6.4 ± 0.3	6.2 ± 0.2	0.60	4.1 ± 0.2	4.5 ± 0.4	0.40	2.5 ± 0.2	1.9 ± 0.1	0.02
Glycine	484 ± 19	536 ± 18	0.053	538 ± 14	639 ± 16	1.8 × 10^−5^	472 ± 27	926 ± 92	9.6 × 10^−4^	485 ± 33	1230 ± 103	3.5 × 10^−6^
Citrulline	91 ± 2	94 ± 5	0.57	77 ± 2	68 ± 2	3.4 × 10^−4^	109 ± 5	102 ± 4	0.30	88 ± 3	75 ± 4	0.02
Arginine	65 ± 4	73 ± 3	0.10	60 ± 2	61 ± 3	0.75	76 ± 8	72 ± 5	0.63	51 ± 5	54 ± 5	0.69
Creatinine	12.1 ± 0.3	12.8 ± 0.3	0.17	11.4 ± 0.3	11.6 ± 0.4	0.60	9.5 ± 0.4	9.2 ± 0.3	0.56	10.6 ± 1.5	8.9 ± 1.4	0.40
Ornithine	139 ± 10	122 ± 6	0.16	115 ± 5	116 ± 7	0.88	119 ± 12	105 ± 10	0.38	158 ± 20	104 ± 10	0.02
Lysine	128 ± 4	135 ± 4	0.24	106 ± 4	106 ± 44	0.98	189 ± 15	194 ± 18	0.84	170 ± 12	139 ± 8	0.03
Histidine	131 ± 4	133 ± 3	0.78	126 ± 2	126 ± 4	0.88	139 ± 8	131 ± 11	0.60	156 ± 12	105 ± 6	1.0 × 10^−3^
Tryptophan	119 ± 5	120 ± 4	0.87	79 ± 3	84 ± 4	0.32	156 ± 17	144 ± 15	0.63	114 ± 11	85 ± 7	0.03
Serine	115 ± 6	125 ± 7	0.32	112 ± 4	127 ± 6	0.06	186 ± 13	218 ± 21	0.26	178 ± 23	167 ± 11	0.67
Proline	150 ± 7	151 ± 4	0.91	129 ± 4	141 ± 8	0.21	241 ± 29	275 ±51	0.63	293 ± 53	202 ± 33	0.14
Valine	104 ± 4	101 ± 3	0.62	102 ± 4	104 ± 4	0.79	136 ± 8	130 ± 11	0.70	147 ± 11	108 ± 6	4.0 × 10^−3^
Phenylalanine	109 ± 3	109 ± 2	0.94	98 ± 2	104 ± 3	0.16	114 ± 12	98 ± 7	0.22	134 ± 18	75 ± 6	3.4 × 10^−3^

Metabolites were measured by mass spectroscopy in plasmas obtained after either a 4 h fast (fasting) or after two hours into the feeding cycle (non-fasting). *p*-values between control and glycine fed groups, separately in males and females and in fasted and non-fasting groups, were derived from 2-sided unpaired *t*-tests. Differences at *p* < 0.05 are highlighted in bold. TMAO, trimethylamine *N*-oxide.

## Data Availability

Individual level data used in the present study are available upon application to the UK Biobank (https://www.ukbiobank.ac.uk/). Summary statistics from the meta-analysis for glycine levels will be posted to a public repository. Summary statistics for glycine levels from all other datasets used in the present study are available through their respective publications. All other relevant data are available upon request from the authors.

## Supplementary Materials

The following supporting information can be downloaded at: https://www.mdpi.com/article/10.3390/nu17010198/s1, Table S1: Clinical Characteristics of Participants in the UK Biobank. Table S2: Description of Datasets Used for GWAS Meta-analysis of Circulating Glycine Levels; Table S3: Results for 61 Loci Significantly Associated with Circulating Glycine Levels; Table S4: PheWAS Results for 61 Loci Significantly Associated with Circulating Glycine Levels; Table S5: Mendelian Randomization Results for Glycine SNPs and CAD Risk Factors; Table S6: Composition of Control and Glycine-supplemented Diets; Table S7: Effect of Glycine Supplementation on Plasma Inflammatory Biomarker Levels; Table S8: Effect of Glycine Supplementation on Blood Cell Traits. Figure S1: Regional plots for 26 novel loci identified for circulating glycine levels in GWAS meta-analysis.

## References

[1] Lusis A.J. (2000). Atherosclerosis. Nature.

[2] Libby P. (2021). The changing landscape of atherosclerosis. Nature.

[3] Bjorkegren J.L.M., Lusis A.J. (2022). Atherosclerosis: Recent developments. Cell.

[4] Aragam K.G., Jiang T., Goel A., Kanoni S., Wolford B.N., Atri D.S., Weeks E.M., Wang M., Hindy G., Zhou W. (2022). Discovery and systematic characterization of risk variants and genes for coronary artery disease in over a million participants. Nat. Genet..

[5] Tcheandjieu C., Zhu X., Hilliard A.T., Clarke S.L., Napolioni V., Ma S., Lee K.M., Fang H., Chen F., Lu Y. (2022). Large-scale genome-wide association study of coronary artery disease in genetically diverse populations. Nat. Med..

[6] Wong N.D., Zhao Y., Quek R.G.W., Blumenthal R.S., Budoff M.J., Cushman M., Garg P., Sandfort V., Tsai M., Lopez J.A.G. (2017). Residual atherosclerotic cardiovascular disease risk in statin-treated adults: The multi-ethnic study of atherosclerosis. J. Clin. Lipidol..

[7] Civelek M., Lusis A.J. (2014). Systems genetics approaches to understand complex traits. Nat. Rev. Genet..

[8] Ding Y., Svingen G.F., Pedersen E.R., Gregory J.F., Ueland P.M., Tell G.S., Nygard O.K. (2016). Plasma glycine and risk of acute myocardial infarction in patients with suspected stable angina pectoris. J. Am. Heart Assoc..

[9] Ding Y., Pedersen E.R., Svingen G.F., Helgeland O., Gregory J.F., Loland K.H., Meyer K., Tell G.S., Ueland P.M., Nygard O.K. (2016). Methylenetetrahydrofolate dehydrogenase 1 polymorphisms modify the associations of plasma glycine and serine with risk of acute myocardial infarction in patients with stable angina pectoris in wenbit3 (western norway b vitamin intervention trial). Circ. Cardiovasc. Genet..

[10] El Hafidi M., Perez I., Banos G. (2006). Is glycine effective against elevated blood pressure?. Curr. Opin. Clin. Nutr. Metab. Care.

[11] Diaz-Flores M., Cruz M., Duran-Reyes G., Munguia-Miranda C., Loza-Rodriguez H., Pulido-Casas E., Torres-Ramirez N., Gaja-Rodriguez O., Kumate J., Baiza-Gutman L.A. (2013). Oral supplementation with glycine reduces oxidative stress in patients with metabolic syndrome, improving their systolic blood pressure. Can. J. Physiol. Pharmacol..

[12] Gannon M.C., Nuttall J.A., Nuttall F.Q. (2002). The metabolic response to ingested glycine. Am. J. Clin. Nutr..

[13] Schemmer P., Zhong Z., Galli U., Wheeler M.D., Xiangli L., Bradford B.U., Conzelmann L.O., Forman D., Boyer J., Thurman R.G. (2013). Glycine reduces platelet aggregation. Amino Acids.

[14] Amin A.M., Sheau Chin L., Teh C.H., Mostafa H., Mohamed Noor D.A., Abdul Kader M., Kah Hay Y., Ibrahim B. (2018). Pharmacometabolomics analysis of plasma to phenotype clopidogrel high on treatment platelets reactivity in coronary artery disease patients. Eur. J. Pharm. Sci..

[15] Jia Q., Han Y., Huang P., Woodward N.C., Gukasyan J., Kettunen J., Ala-Korpela M., Anufrieva O., Wang Q., Perola M. (2019). Genetic determinants of circulating glycine levels and risk of coronary artery disease. J. Am. Heart Assoc..

[16] Wittemans L.B.L., Lotta L.A., Oliver-Williams C., Stewart I.D., Surendran P., Karthikeyan S., Day F.R., Koulman A., Imamura F., Zeng L. (2019). Assessing the causal association of glycine with risk of cardio-metabolic diseases. Nat. Commun..

[17] Chang X., Wang L., Guan S.P., Kennedy B.K., Liu J., Khor C.C., Low A.F., Chan M.Y., Yuan J.M., Koh W.P. (2021). The association of genetically determined serum glycine with cardiovascular risk in east asians. Nutr. Metab. Cardiovasc. Dis..

[18] Hu S., Lin Z., Hu M.J., Tan J.S., Guo T.T., Huang X., Hua L. (2023). Causal relationships of circulating amino acids with cardiovascular disease: A trans-ancestry mendelian randomization analysis. J. Transl. Med..

[19] Wang Z., Yang Q. (2024). The causal relationship between human blood metabolites and the risk of visceral obesity: A mendelian randomization analysis. Lipids Health Dis..

[20] Smith M.L., Bull C.J., Holmes M.V., Davey Smith G., Sanderson E., Anderson E.L., Bell J.A. (2023). Distinct metabolic features of genetic liability to type 2 diabetes and coronary artery disease: A reverse mendelian randomization study. EBioMedicine.

[21] Xie W., Wood A.R., Lyssenko V., Weedon M.N., Knowles J.W., Alkayyali S., Assimes T.L., Quertermous T., Abbasi F., Paananen J. (2013). Genetic variants associated with glycine metabolism and their role in insulin sensitivity and type 2 diabetes. Diabetes.

[22] White P.J., Lapworth A.L., McGarrah R.W., Kwee L.C., Crown S.B., Ilkayeva O., An J., Carson M.W., Christopher B.A., Ball J.R. (2020). Muscle-liver trafficking of bcaa-derived nitrogen underlies obesity-related glycine depletion. Cell Rep..

[23] Tan H.C., Hsu J.W., Tai E.S., Chacko S., Wu V., Lee C.F., Kovalik J.P., Jahoor F. (2022). De novo glycine synthesis is reduced in adults with morbid obesity and increases following bariatric surgery. Front. Endocrinol..

[24] Bycroft C., Freeman C., Petkova D., Band G., Elliott L.T., Sharp K., Motyer A., Vukcevic D., Delaneau O., O’Connell J. (2018). The uk biobank resource with deep phenotyping and genomic data. Nature.

[25] Hartiala J.A., Tang W.H., Wang Z., Crow A.L., Stewart A.F., Roberts R., McPherson R., Erdmann J., Willenborg C., Hazen S.L. (2016). Genome-wide association study and targeted metabolomics identifies sex-specific association of cps1 with coronary artery disease. Nat. Commun..

[26] Bhattacharyya T., Nicholls S.J., Topol E.J., Zhang R., Yang X., Schmitt D., Fu X., Shao M., Brennan D.M., Ellis S.G. (2008). Relationship of paraoxonase 1 (pon1) gene polymorphisms and functional activity with systemic oxidative stress and cardiovascular risk. JAMA.

[27] Hartiala J., Li D., Conti D.V., Vikman S., Patel Y., Tang W.H., Brennan M.L., Newman J.W., Stephensen C.B., Armstrong P. (2011). Genetic contribution of the leukotriene pathway to coronary artery disease. Hum. Genet..

[28] Tang W.H., Hartiala J., Fan Y., Wu Y., Stewart A.F., Erdmann J., Kathiresan S., Roberts R., McPherson R., Allayee H. (2012). Clinical and genetic association of serum paraoxonase and arylesterase activities with cardiovascular risk. Arterioscler. Thromb. Vasc. Biol..

[29] Reiner A.P., Hartiala J., Zeller T., Bis J.C., Dupuis J., Fornage M., Baumert J., Kleber M.E., Wild P.S., Baldus S. (2013). Genome-wide and gene-centric analyses of circulating myeloperoxidase levels in the charge and care consortia. Hum. Mol. Genet..

[30] Hartiala J., Bennett B.J., Tang W.H., Wang Z., Stewart A.F., Roberts R., McPherson R., Lusis A.J., Hazen S.L., Allayee H. (2014). Comparative genome-wide association studies in mice and humans for trimethylamine n-oxide, a proatherogenic metabolite of choline and l-carnitine. Arterioscler. Thromb. Vasc. Biol..

[31] Hartiala J.A., Han Y., Jia Q., Hilser J.R., Huang P., Gukasyan J., Schwartzman W.S., Cai Z., Biswas S., Tregouet D.A. (2021). Genome-wide analysis identifies novel susceptibility loci for myocardial infarction. Eur. Heart J..

[32] United States Renal Data System Usrds 2015 Annual Data Report. http://www.usrds.org/adr.aspx.

[33] Julkunen H., Cichonska A., Tiainen M., Koskela H., Nybo K., Makela V., Nokso-Koivisto J., Kristiansson K., Perola M., Salomaa V. (2023). Atlas of plasma nmr biomarkers for health and disease in 118,461 individuals from the uk biobank. Nat. Commun..

[34] Chang C.C., Chow C.C., Tellier L.C., Vattikuti S., Purcell S.M., Lee J.J. (2015). Second-generation plink: Rising to the challenge of larger and richer datasets. Gigascience.

[35] Loh P.R., Tucker G., Bulik-Sullivan B.K., Vilhjalmsson B.J., Finucane H.K., Salem R.M., Chasman D.I., Ridker P.M., Neale B.M., Berger B. (2015). Efficient bayesian mixed-model analysis increases association power in large cohorts. Nat. Genet..

[36] Kettunen J., Demirkan A., Wurtz P., Draisma H.H., Haller T., Rawal R., Vaarhorst A., Kangas A.J., Lyytikainen L.P., Pirinen M. (2016). Genome-wide study for circulating metabolites identifies 62 loci and reveals novel systemic effects of lpa. Nat. Commun..

[37] Chai J.F., Raichur S., Khor I.W., Torta F., Chew W.S., Herr D.R., Ching J., Kovalik J.P., Khoo C.M., Wenk M.R. (2020). Associations with metabolites in chinese suggest new metabolic roles in alzheimer’s and parkinson’s diseases. Hum. Mol. Genet..

[38] Feofanova E.V., Chen H., Dai Y., Jia P., Grove M.L., Morrison A.C., Qi Q., Daviglus M., Cai J., North K.E. (2020). A genome-wide association study discovers 46 loci of the human metabolome in the hispanic community health study/study of latinos. Am. J. Hum. Genet..

[39] Sakaue S., Kanai M., Tanigawa Y., Karjalainen J., Kurki M., Koshiba S., Narita A., Konuma T., Yamamoto K., Akiyama M. (2021). A cross-population atlas of genetic associations for 220 human phenotypes. Nat. Genet..

[40] Lotta L.A., Pietzner M., Stewart I.D., Wittemans L.B.L., Li C., Bonelli R., Raffler J., Biggs E.K., Oliver-Williams C., Auyeung V.P.W. (2021). A cross-platform approach identifies genetic regulators of human metabolism and health. Nat. Genet..

[41] Chen Y., Lu T., Pettersson-Kymmer U., Stewart I.D., Butler-Laporte G., Nakanishi T., Cerani A., Liang K.Y.H., Yoshiji S., Willett J.D.S. (2023). Genomic atlas of the plasma metabolome prioritizes metabolites implicated in human diseases. Nat. Genet..

[42] Rueger S., McDaid A., Kutalik Z. (2018). Evaluation and application of summary statistic imputation to discover new height-associated loci. PLoS Genet..

[43] Willer C.J., Li Y., Abecasis G.R. (2010). Metal: Fast and efficient meta-analysis of genomewide association scans. Bioinformatics.

[44] Turner S.D. (2018). Qqman: An r package for visualizing gwas results using q-q and manhattan plots. J. Open Source Softw..

[45] Watanabe K., Taskesen E., van Bochoven A., Posthuma D. (2017). Functional mapping and annotation of genetic associations with fuma. Nat. Commun..

[46] Kamat M.A., Blackshaw J.A., Young R., Surendran P., Burgess S., Danesh J., Butterworth A.S., Staley J.R. (2019). Phenoscanner v2: An expanded tool for searching human genotype-phenotype associations. Bioinformatics.

[47] R Core Team (2022). R: A Language and Environment for Statistical Computing.

[48] Hemani G., Tilling K., Davey Smith G. (2017). Orienting the causal relationship between imprecisely measured traits using gwas summary data. PLoS Genet..

[49] Hemani G., Zheng J., Elsworth B., Wade K.H., Haberland V., Baird D., Laurin C., Burgess S., Bowden J., Langdon R. (2018). The mr-base platform supports systematic causal inference across the human phenome. Elife.

[50] Evangelou E., Warren H.R., Mosen-Ansorena D., Mifsud B., Pazoki R., Gao H., Ntritsos G., Dimou N., Cabrera C.P., Karaman I. (2018). Genetic analysis of over 1 million people identifies 535 new loci associated with blood pressure traits. Nat. Genet..

[51] Yengo L., Sidorenko J., Kemper K.E., Zheng Z., Wood A.R., Weedon M.N., Frayling T.M., Hirschhorn J., Yang J., Visscher P.M. (2018). Meta-analysis of genome-wide association studies for height and body mass index in approximately 700000 individuals of european ancestry. Hum. Mol. Genet..

[52] Chen J., Spracklen C.N., Marenne G., Varshney A., Corbin L.J., Luan J., Willems S.M., Wu Y., Zhang X., Horikoshi M. (2021). The trans-ancestral genomic architecture of glycemic traits. Nat. Genet..

[53] Graham S.E., Clarke S.L., Wu K.H., Kanoni S., Zajac G.J.M., Ramdas S., Surakka I., Ntalla I., Vedantam S., Winkler T.W. (2021). The power of genetic diversity in genome-wide association studies of lipids. Nature.

[54] Mahajan A., Spracklen C.N., Zhang W., Ng M.C.Y., Petty L.E., Kitajima H., Yu G.Z., Rueger S., Speidel L., Kim Y.J. (2022). Multi-ancestry genetic study of type 2 diabetes highlights the power of diverse populations for discovery and translation. Nat. Genet..

[55] Mehrabian M., Schulthess F.T., Nebohacova M., Castellani L.W., Zhou Z., Hartiala J., Oberholzer J., Lusis A.J., Maedler K., Allayee H. (2008). Identification of alox5 as a gene regulating adiposity and pancreatic function. Diabetologia.

[56] Woodward N.C., Crow A.L., Zhang Y., Epstein S., Hartiala J., Johnson R., Kocalis H., Saffari A., Sankaranarayanan I., Akbari O. (2019). Exposure to nanoscale particulate matter from gestation to adulthood impairs metabolic homeostasis in mice. Sci. Rep..

[57] Matthews D.R., Hosker J.P., Rudenski A.S., Naylor B.A., Treacher D.F., Turner R.C. (1985). Homeostasis model assessment: Insulin resistance and beta-cell function from fasting plasma glucose and insulin concentrations in man. Diabetologia.

[58] Mehrabian M., Allayee H., Wong J., Shi W., Wang X.P., Shaposhnik Z., Funk C.D., Lusis A.J. (2002). Identification of 5-lipoxygenase as a major gene contributing to atherosclerosis susceptibility in mice. Circ. Res..

[59] Lin C., Sun Z., Mei Z., Zeng H., Zhao M., Hu J., Xia M., Huang T., Wang C., Gao X. (2022). The causal associations of circulating amino acids with blood pressure: A mendelian randomization study. BMC Med..

[60] Wongkittichote P., Ah Mew N., Chapman K.A. (2017). Propionyl-coa carboxylase—A review. Mol. Genet. Metab..

[61] Li H., Lampe J.N. (2019). Neonatal cytochrome p450 cyp3a7: A comprehensive review of its role in development, disease, and xenobiotic metabolism. Arch. Biochem. Biophys..

[62] Ren J., Wang W., Nie J., Yuan W., Zeng A.P. (2022). Understanding and engineering glycine cleavage system and related metabolic pathways for c1-based biosynthesis. Adv. Biochem. Eng. Biotechnol..

[63] Luo M., Willis W.T., Coletta D.K., Langlais P.R., Mengos A., Ma W., Finlayson J., Wagner G.R., Shi C.X., Mandarino L.J. (2019). Deletion of the mitochondrial protein vwa8 induces oxidative stress and an hnf4alpha compensatory response in hepatocytes. Biochemistry.

[64] Laan L., Klar J., Sobol M., Hoeber J., Shahsavani M., Kele M., Fatima A., Zakaria M., Anneren G., Falk A. (2020). DNA methylation changes in down syndrome derived neural ipscs uncover co-dysregulation of znf and hox3 families of transcription factors. Clin. Epigenet..

[65] Shi Y., Yasui M., Hara-Chikuma M. (2022). Aqp9 transports lactate in tumor-associated macrophages to stimulate an m2-like polarization that promotes colon cancer progression. Biochem. Biophys. Rep..

[66] Black M.H., Fingerlin T.E., Allayee H., Zhang W., Xiang A.H., Trigo E., Hartiala J., Lehtinen A.B., Haffner S.M., Bergman R.N. (2008). Evidence of interaction between pparg2 and hnf4a contributing to variation in insulin sensitivity in mexican americans. Diabetes.

[67] Osman W., Hassoun A., Jelinek H.F., Almahmeed W., Afandi B., Tay G.K., Alsafar H. (2020). Genetics of type 2 diabetes and coronary artery disease and their associations with twelve cardiometabolic traits in the united arab emirates population. Gene.

[68] Fathzadeh M., Li J., Rao A., Cook N., Chennamsetty I., Seldin M., Zhou X., Sangwung P., Gloudemans M.J., Keller M. (2020). Fam13a affects body fat distribution and adipocyte function. Nat. Commun..

[69] Loh N.Y., Minchin J.E.N., Pinnick K.E., Verma M., Todorcevic M., Denton N., Moustafa J.E., Kemp J.P., Gregson C.L., Evans D.M. (2020). Rspo3 impacts body fat distribution and regulates adipose cell biology in vitro. Nat. Commun..

[70] Moebius F.F., Fitzky B.U., Wietzorrek G., Haidekker A., Eder A., Glossmann H. (2003). Cloning of an emopamil-binding protein (ebp)-like protein that lacks sterol delta8-delta7 isomerase activity. Biochem. J..

[71] Ahmed H.A., Fadel F.I., Abdel Mawla M.A., Salah D.M., Fathallah M.G., Amr K. (2022). Next-generation sequencing in identification of pathogenic variants in primary hyperoxaluria among 21 egyptian families: Identification of two novel agxt gene mutations. Mol. Genet. Genomic Med..

[72] Leung K.Y., De Castro S.C.P., Galea G.L., Copp A.J., Greene N.D.E. (2021). Glycine cleavage system h protein is essential for embryonic viability, implying additional function beyond the glycine cleavage system. Front. Genet..

[73] Kikuchi G., Motokawa Y., Yoshida T., Hiraga K. (2008). Glycine cleavage system: Reaction mechanism, physiological significance, and hyperglycinemia. Proc. Jpn. Academy. Ser. B Phys. Biol. Sci..

[74] Zaric B.L., Radovanovic J.N., Gluvic Z., Stewart A.J., Essack M., Motwalli O., Gojobori T., Isenovic E.R. (2020). Atherosclerosis linked to aberrant amino acid metabolism and immunosuppressive amino acid catabolizing enzymes. Front. Immunol..

[75] Spracklen C.N., Horikoshi M., Kim Y.J., Lin K., Bragg F., Moon S., Suzuki K., Tam C.H.T., Tabara Y., Kwak S.H. (2020). Identification of type 2 diabetes loci in 433,540 east asian individuals. Nature.

[76] Buniello A., MacArthur J.A.L., Cerezo M., Harris L.W., Hayhurst J., Malangone C., McMahon A., Morales J., Mountjoy E., Sollis E. (2019). The nhgri-ebi gwas catalog of published genome-wide association studies, targeted arrays and summary statistics 2019. Nucleic Acids Res..

[77] Chen M.H., Raffield L.M., Mousas A., Sakaue S., Huffman J.E., Moscati A., Trivedi B., Jiang T., Akbari P., Vuckovic D. (2020). Trans-ethnic and ancestry-specific blood-cell genetics in 746,667 individuals from 5 global populations. Cell.

[78] Nakatochi M., Kanai M., Nakayama A., Hishida A., Kawamura Y., Ichihara S., Akiyama M., Ikezaki H., Furusyo N., Shimizu S. (2019). Genome-wide meta-analysis identifies multiple novel loci associated with serum uric acid levels in japanese individuals. Commun. Biol..

[79] Bowden J., Davey Smith G., Burgess S. (2015). Mendelian randomization with invalid instruments: Effect estimation and bias detection through egger regression. Int. J. Epidemiol..

[80] Schmidt A.F., Dudbridge F. (2018). Mendelian randomization with egger pleiotropy correction and weakly informative bayesian priors. Int. J. Epidemiol..

[81] Alves A., Bassot A., Bulteau A.L., Pirola L., Morio B. (2019). Glycine metabolism and its alterations in obesity and metabolic diseases. Nutrients.

[82] Tan H.C., Hsu J.W., Tai E.S., Chacko S., Kovalik J.P., Jahoor F. (2024). The impact of obesity-associated glycine deficiency on the elimination of endogenous and exogenous metabolites via the glycine conjugation pathway. Front. Endocrinol..

[83] Razquin C., Ruiz-Canela M., Toledo E., Clish C.B., Guasch-Ferre M., Garcia-Gavilan J.F., Wittenbecher C., Alonso-Gomez A., Fito M., Liang L. (2022). Circulating amino acids and risk of peripheral artery disease in the predimed trial. Int. J. Mol. Sci..

[84] Rom O., Liu Y., Finney A.C., Ghrayeb A., Zhao Y., Shukha Y., Wang L., Rajanayake K.K., Das S., Rashdan N.A. (2022). Induction of glutathione biosynthesis by glycine-based treatment mitigates atherosclerosis. Redox Biol..

[85] Rom O., Liu Y., Liu Z., Zhao Y., Wu J., Ghrayeb A., Villacorta L., Fan Y., Chang L., Wang L. (2020). Glycine-based treatment ameliorates nafld by modulating fatty acid oxidation, glutathione synthesis, and the gut microbiome. Sci. Transl. Med..

[86] El-Hafidi M., Franco M., Ramirez A.R., Sosa J.S., Flores J.A.P., Acosta O.L., Salgado M.C., Cardoso-Saldana G. (2018). Glycine increases insulin sensitivity and glutathione biosynthesis and protects against oxidative stress in a model of sucrose-induced insulin resistance. Oxid. Med. Cell Longev..

[87] Ceron E., Bernal-Alcantara D., Vanda B., Sommer B., Gonzalez-Trujano E., Alvarado-Vasquez N. (2021). Glycine supplementation during six months does not alter insulin, glucose or triglyceride plasma levels in healthy rats. Int. J. Vitam. Nutr. Res..

