# Supplementary material for: Exploring the Role of Glycine Metabolism in Coronary Artery Disease: Insights from Human Genetics and Mouse Models
Source: Nutrients. 2025 Jan 6;17(1):198. doi: 10.3390/nu17010198 (PMC11723402; doi:10.3390/nu17010198)
Supplement: Supplementary file 1 [file nutrients-17-00198-s001.zip › nutrients-3357475 Updated Supplemental Figure S1 .pdf]

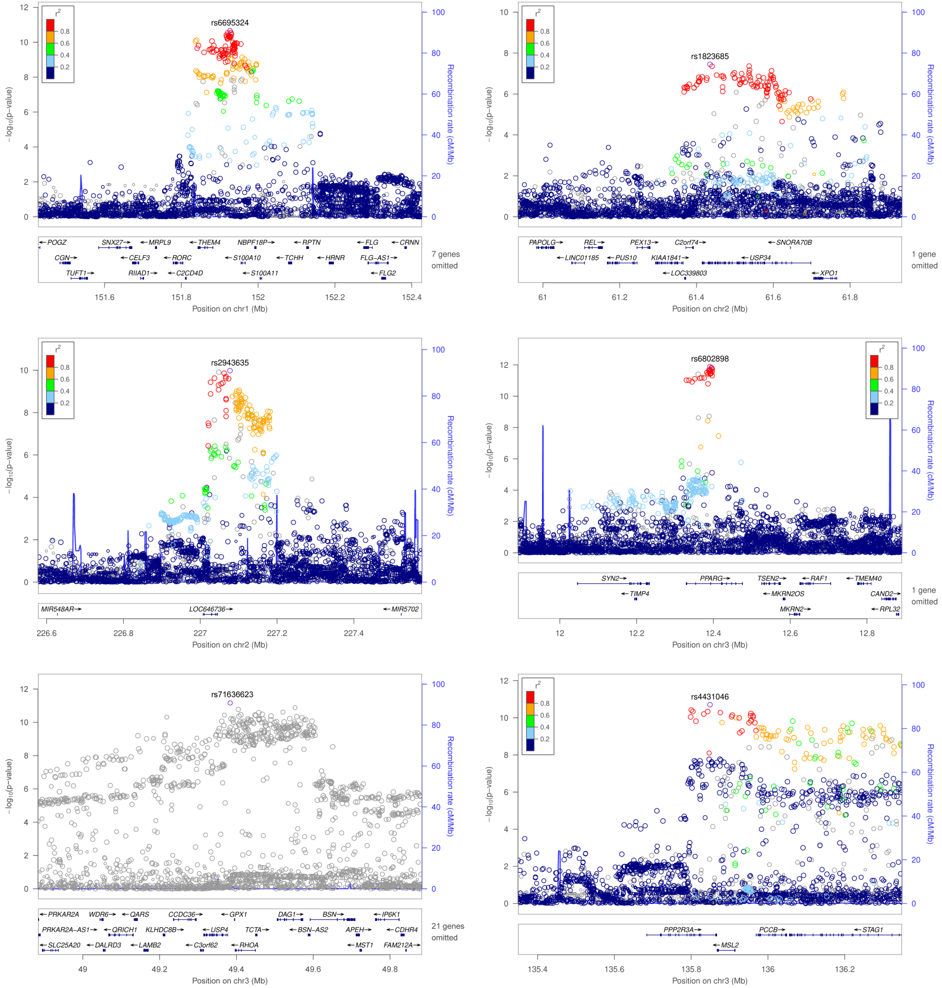
rs6695324
r2
0.8
0.6
0.4
0.2
−log10(p−value)
Recombination rate (cM/Mb)
POGZ
SNX27
MRPL9
THEM4
NBPF18P
RPTN
FLG
CRNN
CGN
CELF3
RORC
S100A10
TCHH
HRNR
FLG−AS1
TUFT1
RIIAD1
C2CD4D
S100A11
FLG2
7 genes omitted
Position on chr1 (Mb)
rs1823685
PAPOLG
REL
PEX13
C2orf74
SNORA70B
LINC01185
PUS10
KIAA1841
USP34
LOC339803
XPO1
1 gene omitted
Position on chr2 (Mb)
rs2943635
MIR548AR
LOC646736
MIR5702
Position on chr2 (Mb)
rs6802898
SYN2
PPARG
TSEN2
RAF1
TMEM40
TIMP4
MKRN2OS
CAND2
MKRN2
RPL32
1 gene omitted
Position on chr3 (Mb)
rs71636623
PRKAR2A
WDR6
QARS
CCDC36
GPX1
DAG1
BSN
IP6K1
PRKAR2A−AS1
QRICH1
KLHDC8B
USP4
TCTA
BSN−AS2
APEH
CDHR4
SLC25A20
DALRD3
LAMB2
C3orf62
RHOA
MST1
FAM212A
21 genes omitted
Position on chr3 (Mb)
rs4431046
PPP2R3A
PCCB
STAG1
MSL2
Position on chr3 (Mb)

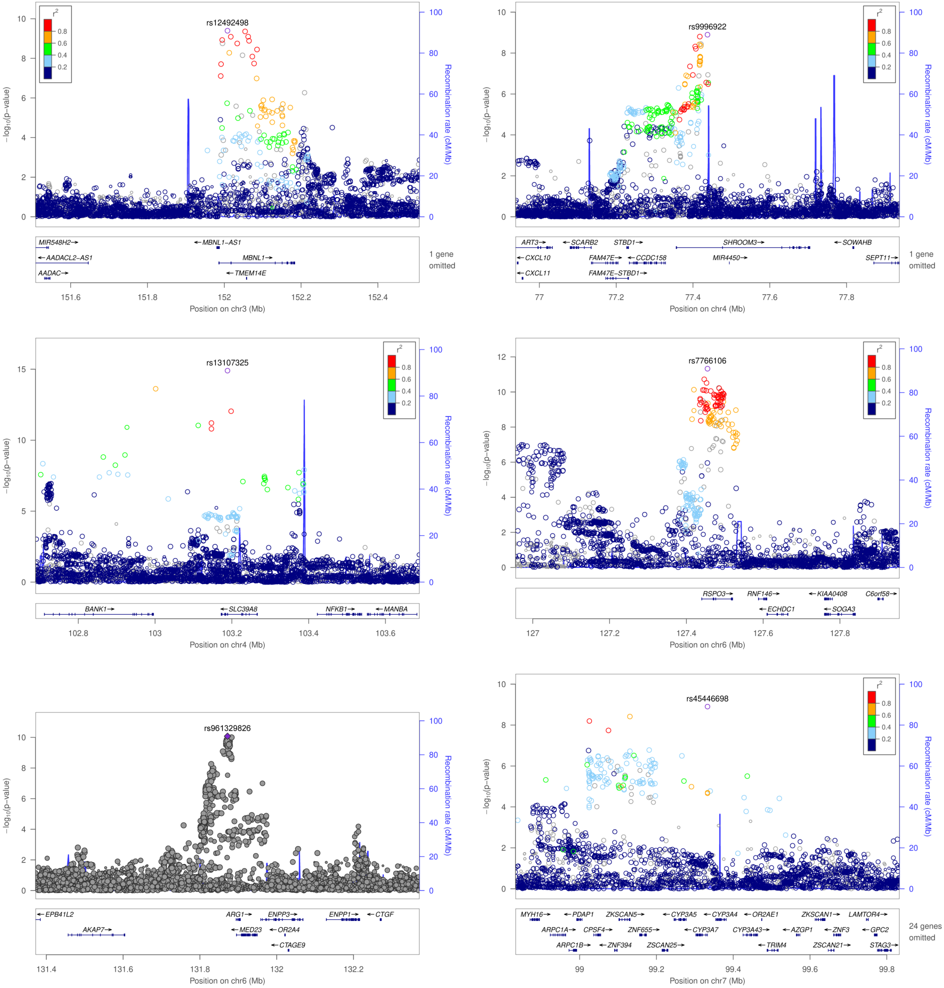
rs12492498
rs9996922
rs13107325
rs7766106
rs961329826
rs45446698
r2
0.8
0.6
0.4
0.2
−log10(p−value)
Recombination rate (cM/Mb)
Position on chr3 (Mb)
Position on chr4 (Mb)
Position on chr4 (Mb)
Position on chr6 (Mb)
Position on chr6 (Mb)
Position on chr7 (Mb)
MIR548H2→
←AADACL2-AS1
AADAC→
←MBNL1-AS1
MBNL1→
←TMEM14E
1 gene omitted
ART3→
←CXCL10
←CXCL11
←SCARB2
STBD1→
FAM47E→
←CCDC158
FAM47E-STBD1→
SHROOM3→
MIR4450→
←SOWAHB
SEPT11→
1 gene omitted
BANK1→
←SLC39A8
NFKB1→
←MANBA
RSPO3→
RNF146→
←ECHDC1
←KIAA0408
←SOGA3
C6orf58→
←EPB41L2
AKAP7→
ARG1→
←MED23
ENPP3→
←OR2A4
←CTAGE9
ENPP1→
←CTGF
MYH16→
ARPC1A→
ARPC1B→
←PDAP1
CPSF4→
←ZNF394
ZKSCAN5→
ZNF655→
ZSCAN25→
←CYP3A5
←CYP3A7
←CYP3A4
←OR2AE1
CYP3A43→
←TRIM4
←AZGP1
ZKSCAN1→
←ZNF3
ZSCAN21→
LAMTOR4→
←GPC2
STAG3→
24 genes omitted

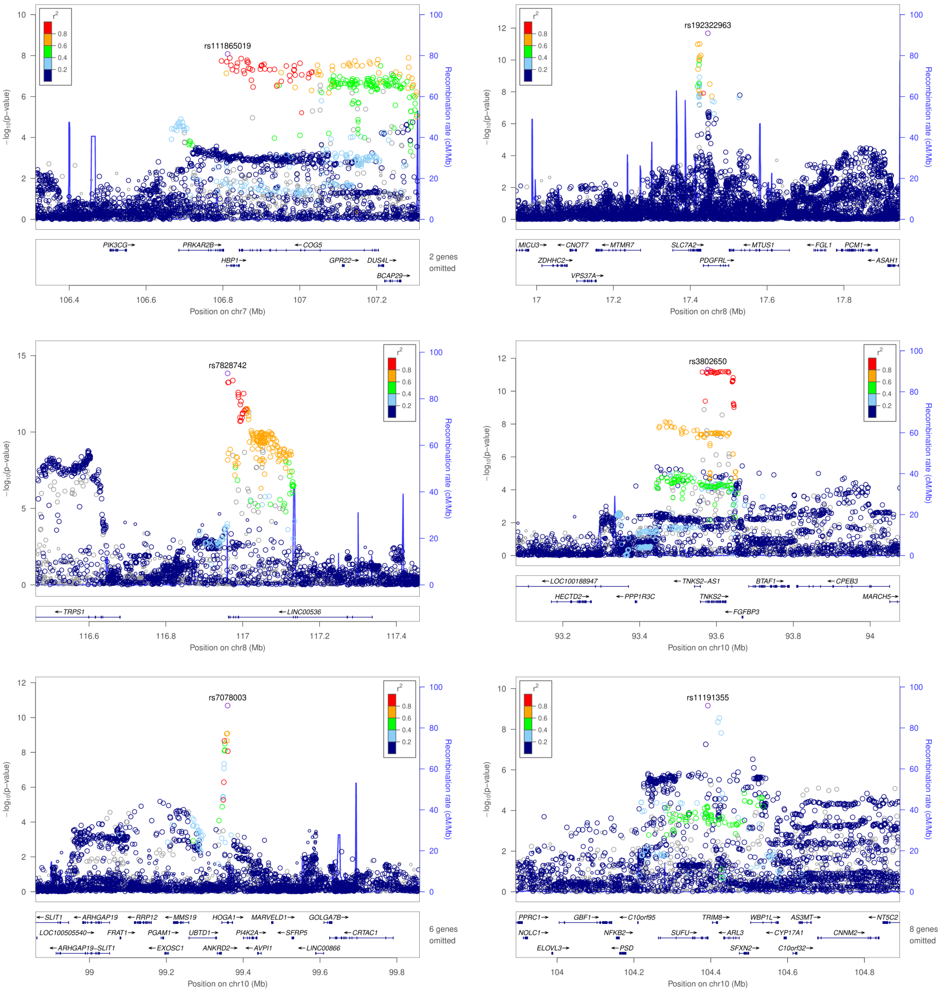

rs111865019
r²
0.8
0.6
0.4
0.2
−log₁₀(p−value)
Recombination rate (cM/Mb)
PIK3CG→
PRKAR2B→
←COG5
HBP1→
GPR22→
DUS4L→
BCAP29→
2 genes omitted
Position on chr7 (Mb)
rs192322963
MICU3→
←CNOT7
←MTMR7
SLC7A2→
←MTUS1
←FGL1
PCM1→
ZDHHC2→
PDGFRL→
←ASAH1
VPS37A→
Position on chr8 (Mb)
rs7828742
←TRPS1
←LINC00536
Position on chr8 (Mb)
rs3802650
←LOC100188947
←TNKS2−AS1
BTAF1→
←CPEB3
HECTD2→
←PPP1R3C
TNKS2→
MARCH5→
←FGFBP3
Position on chr10 (Mb)
rs7078003
←SLIT1
←ARHGAP19
←RRP12
←MMS19
HOGA1→
MARVELD1→
GOLGA7B→
LOC100505540→
FRAT1→
PGAM1→
UBTD1→
PI4K2A→
←SFRP5
←CRTAC1
←ARHGAP19−SLIT1
←EXOSC1
ANKRD2→
←AVPI1
←LINC00866
6 genes omitted
Position on chr10 (Mb)
rs11191355
PPRC1→
GBF1→
←C10orf95
TRIM8→
WBP1L→
AS3MT→
←NT5C2
NOLC1→
NFKB2→
SUFU→
←ARL3
←CYP17A1
CNNM2→
ELOVL3→
←PSD
SFXN2→
C10orf32→
8 genes omitted
Position on chr10 (Mb)

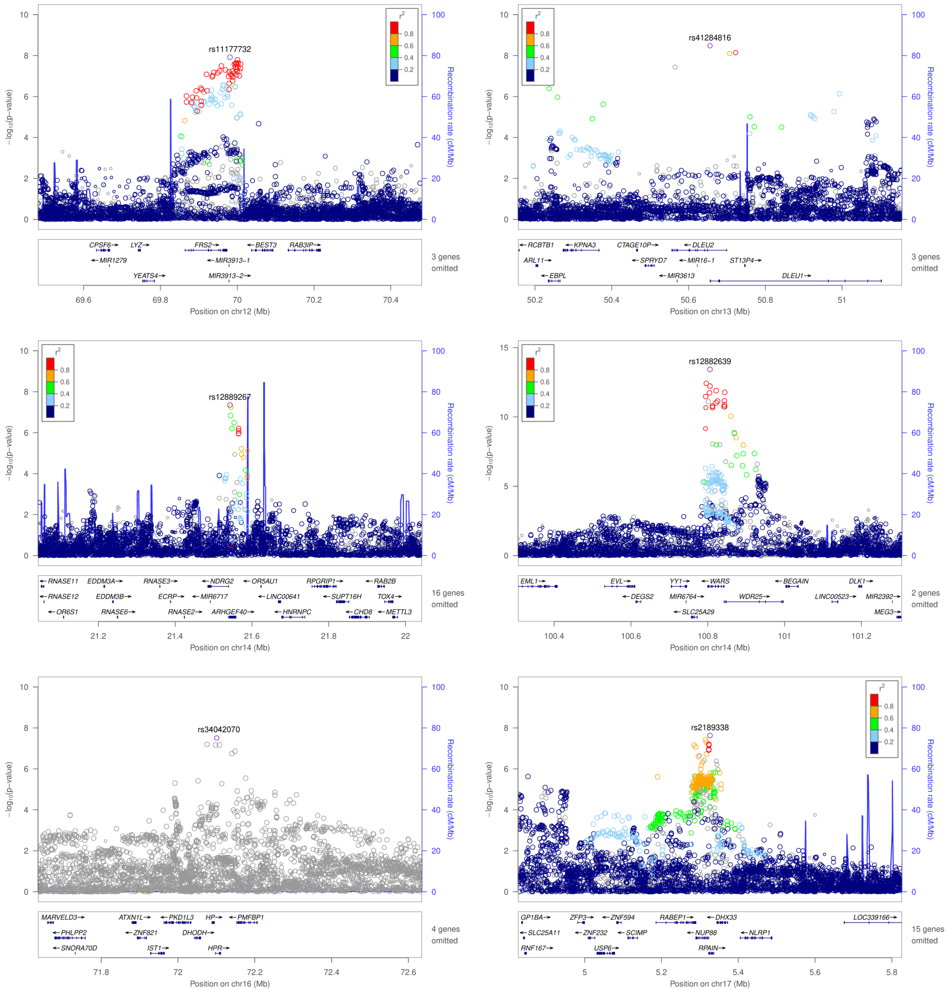
rs11177732
−log10(p−value)
Recombination rate (cM/Mb)
r2
0.8
0.6
0.4
0.2
CPSF6→
LYZ→
FRS2→
←BEST3
RAB3IP→
←MIR1279
←MIR3913−1
YEATS4→
MIR3913−2→
3 genes omitted
Position on chr12 (Mb)
rs41284816
←RCBTB1
←KPNA3
CTAGE10P→
←DLEU2
ARL11→
←SPRYD7
←MIR16−1
ST13P4→
←EBPL
←MIR3613
DLEU1→
3 genes omitted
Position on chr13 (Mb)
rs12889267
←RNASE11
EDDM3A→
RNASE3→
←NDRG2
←OR5AU1
RPGRIP1→
←RAB2B
←RNASE12
EDDM3B→
ECRP→
←MIR6717
←LINC00641
←SUPT16H
TOX4→
←OR6S1
RNASE6→
RNASE2→
ARHGEF40→
←HNRNPC
←CHD8
←METTL3
16 genes omitted
Position on chr14 (Mb)
rs12882639
EML1→
EVL→
YY1→
←WARS
←BEGAIN
DLK1→
←DEGS2
MIR6764→
WDR25→
LINC00523→
MIR2392→
←SLC25A29
MEG3→
2 genes omitted
Position on chr14 (Mb)
rs34042070
MARVELD3→
ATXN1L→
←PKD1L3
HP→
←PMFBP1
←PHLPP2
←ZNF821
DHODH→
←SNORA70D
IST1→
HPR→
4 genes omitted
Position on chr16 (Mb)
rs2189338
GP1BA→
ZFP3→
←ZNF594
RABEP1→
←DHX33
LOC339166→
←SLC25A11
←ZNF232
←SCIMP
←NUP88
←NLRP1
RNF167→
USP6→
RPAIN→
15 genes omitted
Position on chr17 (Mb)

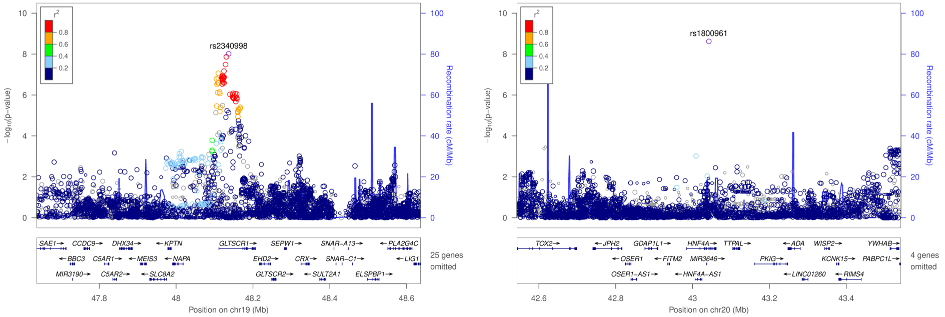

Supplemental Figure S1. Regional plots for 26 novel loci identified for circulating glycine levels in GWAS meta-analysis.
